# Black Carbon Impacts on *Paraburkholderia xenovorans* Strain LB400 Cell Enrichment and Activity: Implications toward Lower-Chlorinated Polychlorinated Biphenyls Biodegradation Potential

**DOI:** 10.1021/acs.est.3c09183

**Published:** 2024-02-15

**Authors:** Qin Dong, Gregory H. LeFevre, Timothy E. Mattes

**Affiliations:** † Department of Civil and Environmental Engineering, University of Iowa, 4105 Seamans Center, Iowa City, Iowa 52242, United States; ‡ IIHR—Hydroscience and Engineering, University of Iowa, 100 C. Maxwell Stanley Hydraulics Laboratory, Iowa City, Iowa 52242, United States

**Keywords:** *Paraburkholderia xenovorans* strain LB400, corn kernel biochar, GAC, biphenyl dioxygenase gene expression, biofilms

## Abstract

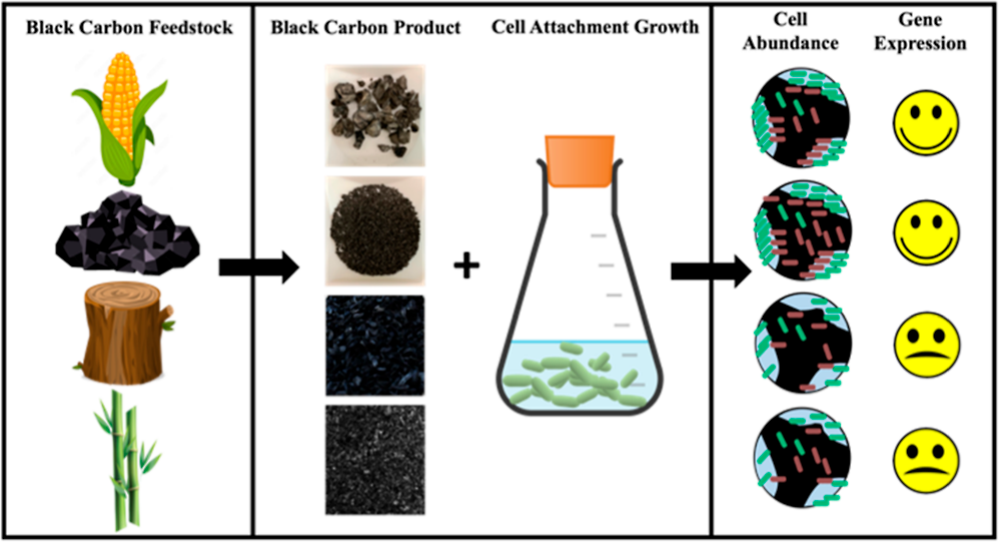

Volatilization of lower-chlorinated polychlorinated biphenyls (LC-PCBs) from sediment poses health threats to nearby communities and ecosystems. Biodegradation combined with black carbon (BC) materials is an emerging bioaugmentation approach to remove PCBs from sediment, but development of aerobic biofilms on BC for long-term, sustained LC-PCBs remediation is poorly understood. This work aimed to characterize the cell enrichment and activity of biphenyl- and benzoate-grown *Paraburkholderia xenovorans* strain LB400 on various BCs. Biphenyl dioxygenase gene (*bphA*) abundance on four BC types demonstrated corn kernel biochar hosted at least 4 orders of magnitude more attached cells per gram than other feedstocks, and microscopic imaging revealed the attached live cell fraction was >1.5× more on corn kernel biochar than GAC. BC characteristics (i.e., sorption potential, pore size, pH) appear to contribute to cell attachment differences. Reverse transcription qPCR indicated that BC feedstocks significantly influenced *bphA* expression in attached cells. The *bphA* transcript-per-gene ratio of attached cells was >10-fold more than suspended cells, confirmed by transcriptomics. RNA-seq also demonstrated significant upregulation of biphenyl and benzoate degradation pathways on attached cells, as well as revealing biofilm formation potential/cell–cell communication pathways. These novel findings demonstrate aerobic PCB-degrading cell abundance and activity could be tuned by adjusting BC feedstocks/attributes to improve LC-PCBs biodegradation potential.

## Abstract 2

LB400 cells attached to corn kernel biochar were more abundant, viable, and active than LB400 attached to other black carbons.

## Introduction

Polychlorinated biphenyls (PCBs) are chemically stable and strongly hydrophobic organic pollutants that persist in sediments.^1−3^ Dissolution and volatilization of semivolatile lower chlorinated PCBs (LC-PCBs)^1,4−8^ from sediment leads to bioaccumulation in the aquatic food chain and human inhalation exposure, which have been linked to toxic effects such as carcinogenesis, neurotoxicity, impaired memory, and endocrine disruption.^9−11^ Although dredging is common practice to decrease total PCB mass in sediment,^12,13^ dredging is costly and resuspension exacerbates LC-PCB release from sediment into the water column.^14−16^ To manage the bioavailability and mobility of PCBs, in situ activated carbon (AC) amendment has been employed to transfer PCBs from sediment to AC particles;^17−21^ however, total contaminant mass load in the environment remains unchanged. Bioremediation is thus required to not only decrease the accessibility of PCBs but also decrease the long-term exposure risk.

Natural bioremediation processes are typically slow; therefore, bioaugmentation has been used to accelerate PCB mass removal in sediments by introducing higher functional cell abundance. A variety of aerobes can degrade PCBs,^22−25^ but *Paraburkholderia xenovorans* strain LB400 demonstrates higher degradation efficacy for wide ranges of PCBs (mono- to hexa-chlorinated biphenyls).^24,26−29^ PCB oxidation proceeds via the *bph* pathway; thus, *bphA* is commonly used as a biomarker because it encodes biphenyl dioxygenase, the initial catabolic enzyme in the pathway.^26,30,31^ Specific carbon/energy sources (e.g., biphenyl, benzoate) are required for *bph* pathway induction, and many PCB congeners with 6 chlorines or less are cometabolized in the presence of biphenyl or benzoate.^26,32−35^ Bioaugmenting sediments with suspended LB400 cells can efficiently oxidize LC-PCB congeners (up to trichlorinated homologues) and mitigate flux to the air.^36,37^ Nevertheless, free cells often demonstrate unsatisfactory survivability and activity due to environmental fluctuations and nutrient availability^38−41^—factors that can be moderated when cells exist as biofilms. Biofilms display increased gene expression levels and produce exopolysaccharides that improve stress tolerance and cell removal resistance.^38,42−45^ Multiple studies have examined immobilized anaerobes that dechlorinate PCBs,^38,46−49^ but comparatively few have focused on removing LC-PCBs through bioaugmentation of immobilized PCB-degrading aerobes to mitigate the direct human exposure pathway.^50−52^ Improved LC-PCB bioremediation strategies require an enhanced understanding of immobilized PCB-degrading cells and how cells interact with surface materials.

Sorptive black carbon (BC) materials (e.g., AC and biochar) have been applied as carriers to deliver microbes for in situ bioremediation because their porous structure can adsorb contaminants, harbor microbes, and facilitate redox reactions between cells and contaminants.^53−55^ For example, aerobic and anaerobic PCB-degrading bacteria have been combined with AC in sediment and porewater, resulting in substantial PCB mass loss.^46,56,57^ Nevertheless, the high AC sorption capacity could decrease nutrient and contaminant bioavailability, and increase environmental stress on microorganisms.^47,48,50,55−58^ Cell attachment to and growth on biochar are reportedly more efficient than with AC because flatter biochar surfaces can beneficially accommodate more cell growth,^38,54^ and microbial redox activity could be enhanced with biochar addition;^55^ however, these processes are poorly understood. Thus, there is a critical need to probe the potential of biochar with beneficial characteristics for cell–surface interaction to aid bacterial growth and microbial activity in bioaugmentation applications, which could be the key to sustained bioremediation. In addition, using BC for pollutant remediation could provide ancillary benefits for long-term climate change mitigation, as BC can facilitate organic carbon accumulation to surface soil of coastal ecosystems.^59,60^

The objective of this study was to characterize the cell enrichment and activity of the model aerobic PCB-degrading strain LB400 on multiple BC surfaces related to aerobic bioremediation of LC-PCBs. By probing BC characteristics beneficial for cell attachment, we gain critical insights concerning interactions between cells and the BC surface. Using LB400 grown on either biphenyl or benzoate as carbon and energy sources, we characterized the cell viability and activity (gene expression and biodegradation performance) for a suite of BC materials using comprehensive microscopic, molecular, and chemical techniques. We investigated microbial biodegradation potential using multiple lines of evidence (i.e., functional gene quantification and expression, transcriptomic sequencing) and assessed the microbial activity status by connecting gene expression assays with microscopic imaging. Finally, we demonstrated approaches to develop denser cell attachment and supply carbon sources that could be useful for developing novel, tunable in situ bioremediation strategies to mitigate LC-PCB exposure to the public.

## Methods and Materials

### Chemicals and Black Carbon Materials

Biphenyl (99.5+%) and sodium benzoate (98.0+%) were purchased from TCI America (Portland, OR). Four types of black carbon (BC) materials were selected to evaluate their impacts on cell attachment. Bamboo and wood biochars were chosen because of their previously demonstrated properties, such as biofilm formation, electron shuttling, adsorption, and reusability of immobilized cells.^57,61−65^ Activated carbon is a BC with high sorption potential that has been commonly used in contaminated site remediation for pollutant sorption and bioaugmentation,^46,48,50^ while corn biochar is an emerging, locally available feedstock that demonstrated improved cell attachment compared to activated carbon in preliminary studies. All BCs tested have porous structure and potential for bacterial colonization but have different surface areas, sorption capacities, and surface structures (Table 1). Corn kernel biochar was obtained from Dr. Albert Ratner’s research group, including two granule sizes (Table 1): small size corn kernel biochar (SB) and large size corn kernel biochar (LB).^66^ Bamboo biochar (BB) was from Lewis Bamboo (Oakman, AL), wood biochar (WB) from Plantonix (Ashland, OR), and granular activated carbon (GAC, Filtrasorb 200) from Calgon Carbon (Pittsburgh, PA). SB, WB, and BB were ground by mortar and pestle and sieved (sieve #20 to #40; 0.4–0.841 mm). The BC granule size range was kept consistent to avoid potentially confounding physical effects (e.g., surface area); however, the effects of two different corn kernel biochar granule sizes (LB size <1.5 cm) on cell attachment were compared. BCs were rinsed with DI water, autoclaved (25 min at 121 °C), and dried (104 °C). BC characteristics were measured by the University of Iowa MATFab Facility. The Brunauer–Emmett–Teller (BET) surface area, pore size distribution, and total porous volume were measured with a Quantachrome Nova 4200e and analyzed with NovaWin software. Solution pH was measured by pH meter (Accumet AR50, Fisher Scientific) after each BC was mixed with 50 mL DI water for 24 h.

**Table 1:** Solid Water Partitioning Coefficient (*K*_d_) for Benzoate and Biphenyl Sorbed on Black Carbons, Mesoporous Volume, Mesopore Size, BET Surface Area, Total Pore Volume, and Granule Size of Each Black Carbon Material^a^

samples	benzoate *K*_d_ (L/g)	biphenyl *K*_d_ (L/g)	mesoporous volume (cm^3^/g)	mesopore size (diameter, nm)	BET surface area (m^2^/g)	total porous volume (cm^3^/g)	granule size (mm)
GAC	1.64 × 10^–1^	1.54 × 10^–1^	0.50	3.76	386	4.82 × 10^–1^	0.4–1.68
WB	4.76 × 10^–2^	1.49 × 10^–1^	0.69	3.74	453	3.28 × 10^–1^	0.4–0.84
BB	4.24 × 10^–2^	8.97 × 10^–2^	0.32	3.80	177	1.40 × 10^–1^	0.4–0.84
SB	1.06 × 10^–3^	8.34 × 10^–3^	0.028	3.23	3	5.66 × 10^–3^	0.4–0.84
LB	1.73 × 10^–3^	1.91 × 10^–5^	0.061	3.21	11	4.17 × 10^–2^	<150

a Mesopore volume and size for SB were measured at 200 °C, while mesopore volume and size for LB, BB, WB, and GAC were measured at room temperature (∼22 °C).

### Bacterial Growth Conditions, and Preparation of BC-Attached Cells

Frozen aerobic PCB-degrading *P. xenovorans* strain LB400 cells were thawed at 4 °C and washed three times with K1 medium^37^ (recipe in Supporting Information S1.1) to remove glycerol. Washed cells (100 μL) were inoculated into 250 mL of K1 medium containing biphenyl crystal or sodium benzoate, where 5 mM biphenyl crystal or 10 mM sodium benzoate was added based on equivalent theoretical oxygen demand (Supporting Information S1.1). Both biphenyl and benzoate are well-established to induce the *bph* pathway in LB400;^26,34,67^ benzoate is more water-soluble and less toxic than biphenyl.^68,69^ When cultures reached midexponential phase (i.e., optical density at 600 nm [OD_600_] = 0.5–0.6), they were concentrated by centrifugation (5000*g*) for 15 min, washed twice with sterile K1 medium, and resuspended overnight with shaking at 150 rpm.^37^

For BC-attached LB400 cell growth, three mL of concentrated LB400 (OD_600_ = 1; 2 × 10^9^ cells/mL) were added to flasks containing K1 medium (27 mL) and a specific BC (0.9 g) in treatment groups. Either 43 mg of sodium benzoate (10 mM) or 22.8 mg of biphenyl (crystal, 5 mM) was added as the sole carbon/energy source. Control groups (no biomass added) were employed to quantify background bacterial contamination. Flasks were shaken at 150 rpm for 10 days. An incubation period of 10 days was chosen based on prior studies^46,48,50,51^ and because a 10 day incubation resulted in higher *bphA* abundances in both suspended and attached cells compared to other incubation periods (Supporting Information S1.1; Figure S1). All of the conditions were conducted in duplicate.

### Viability of LB400 Cells on BCs

Cells attached to BCs were stained with SYTO9 and propidium iodide for 15 min, rinsed, and then covered with 90% glycerol after rinsing (details in Supporting Information S1.2). The distribution of live and dead LB400 cells on BCs was observed by confocal laser scanning microscopy (CLSM, Supporting Information S1.2) with a Leica SP8 STED Super Resolution Confocal (Leica Microsystems, Exton, PA). Imaris 9.2 image analysis software was used to quantify live and dead cells, and cell distance to BC surfaces. Scanning electron microscopy (SEM) was also used to observe cell attachment on BCs (Supporting Information S1.2) using a Hitachi S-4800 (Tokyo, Japan). BC without cells was used as a baseline control.

### Nucleic Acid Extraction, qPCR, and Reverse Transcription (RT)-qPCR

Aqueous samples were separated by pipetting all liquid lying above BCs, and the remaining BCs were retrieved using sterile inoculating loops (Thermo Fisher Scientific, Waltham, MA). Aqueous DNA sample extraction for qPCR was performed with a modified DNeasy PowerWater Sterivex Kit (Qiagen, Carol Stream, Illinois) protocol that bypasses the Sterivex filtration step to accommodate small sample volumes (described in Supporting Information S1.3).^70^ For BC samples, DNA for qPCR was extracted with a Qiagen DNeasy PowerSoil Pro Kit. RNA and DNA for RT-qPCR and RNA-seq were extracted with the Qiagen RNeasy PowerSoil Total RNA kit in series with the Qiagen RNeasy PowerSoil DNA Elution kit to minimize bias from the extraction steps.

Luciferase control mRNA (1 ng = 6.08 × 10^9^ transcripts; Promega, Madison, WI) was added to RNA extracts after cell lysis as an internal standard.^71,72^ DNA contamination was removed from RNA extracts in 20 μL reactions containing 10 μL of RNA and 4U DNase I (Thermo Fisher Scientific, Waltham, MA). RNA extracts were stored at −80 °C prior to RT or control qPCRs. RNA was reverse-transcribed to cDNA in 20 μL reactions (containing 11 μL of RNA, 50 ng of random hexamer, 10 mM dNTPs) using the SuperScript IV First-Strand Synthesis System (Invitrogen, Carlsbad, CA). Qubit dsDNA HS and RNA HS Assay kits were used to quantify DNA and RNA concentrations (Table S1) with the Qubit 4 fluorometer (Thermo Fisher Scientific).

The *bphA* gene abundance (DNA) and *bphA* and luciferase transcript abundance (cDNA) were measured by qPCR using primer sets *bphA* 463*f*/674*r* and *ref* forward primer/*ref* reverse primer (nucleotide positions 1691/1758), respectively (Table S2).^72,73^ Detailed qPCR and QA/QC procedures are described in Supporting Information S1.4. Additional qPCR information is provided in Table S3 to follow MIQE guidelines.^74^ Residual *bphA* DNA measured in RNA controls was at least 4 orders of magnitude lower than in the samples (Figure S2). Normality tests of all DNA and cDNA data indicated that ∼90% were not significantly different from a normal distribution (*p* > 0.05, Figures S3–S5).

The mRNA recovery efficiency (i.e., the ratio of measured luciferase cDNA abundance to luciferase transcripts added), was used to correct transcript abundance estimates for RT efficiency and mRNA losses during extraction and DNA digestion,^72^ but is not intended to account for absolute mRNA recovery. Transcript abundance was normalized by the gene abundance to determine the transcript per gene ratio. The mRNA recovery efficiency ranged from 0.78 to 1.22% (Figure S6), where the efficiency is similar to other reports with most of the mRNA loss occurring during DNA digestion and RT.^72,75^

### RNA-seq, Transcriptome Sequence Processing, and Analysis

RNA extracted from suspended and attached LB400 cells in SB, LB, and GAC, was purified and concentrated with the RNA Clean & Concentrator kit (Zymo Research, Irvine, California). RNA and DNA concentrations were measured by Nanodrop and Qubit for quality control. Custom LB400 probes for rRNA depletion were obtained from Integrated DNA Technologies (Coralville, Iowa). RNA-seq libraries were prepared with the Illumina TruSeq stranded Total RNA prep kit, loaded at equal concentration on an Illumina NovaSeq 6000 SP flow cell, and sequenced (PE100) at the University of Iowa, Iowa Institute of Human Genetics (IIHG). Twelve samples were sequenced, yielding between 1.62 × 10^7^ and 2.07 × 10^7^ reads per sample.

Raw sequence data (fastq files) were processed online with the Galaxy server (usegalaxy.org). Sequence read quality was checked by FastQC.^76^ Fastq files were trimmed and filtered with fastp (0.53–0.78% of total reads filtered in each sample).^77^ Trimmed paired-end reads were joined with Fastq interlacer and mapped to the *P. xenovorans* strain LB400 genome (Genbank accession nos. NC_007951–NC_007953) using HISAT2 (62.56–98.12% of filtered reads mapped per treatment).^78^ HISAT2-generated BAM files were used to generate transcript count tables with FeatureCount.^79^ Differential gene expression analysis among BC-attached cells and between BC-attached cells and suspended cells was performed with DESeq2.^80^ Differentially expressed genes at the 95% confidence level (*p*-adj <0.05) were considered for further analysis. Additional RNA-seq data analysis parameters are described in Supporting Information S1.4.

### Carbon Source Biodegradation Assays with BC-Attached Cells, and Sorption of Benzoate and Biphenyl to BCs

Biodegradation of acetate and benzoate was measured over time to evaluate the activity of the LB400 cells attached to BCs. Acetate is readily metabolized by LB400 via the TCA cycle; thus, biodegradation of acetate was the first indicator that viable and active cells were attached to black carbon. In contrast, benzoate is aromatic and induces the *bph* pathway;^67^ thus benzoate undergoes similar ring cleavage to PCBs and indicates PCB biodegradation potential. Both acetate and benzoate are easily measurable and can avoid the complications associated with hydrophobic properties of PCBs in terms of low solubility and high tendency to sorb onto BCs.^68,81^ BCs (SB or GAC) with attached LB400 cells were weighed in sterile 5 mL centrifuge tubes and washed three times with fresh K1 medium before transferring to new batches. Acetate (3 mM) or benzoate (10 mM) was added to 30 mL of fresh K1 medium containing 0.6 g (wet weight) of BC + attached cells. Sterile BC controls (no attached LB400 cells) with carbon sources accounted for sorption losses. Benzoate and acetate analysis methods are described in Supporting Information S1.6. Liquid samples were collected daily and filtered (0.22 μm PVDF) prior to analysis. Duplicates were used for each treatment and the control group. The solid–water partitioning coefficient (*K*_d_) was quantified for all four BCs to compare sorption behavior (Supporting Information S1.5; Figure S7). Biphenyl extraction and analysis methods are described in Supporting Information S1.6 and Figure S8.

### Soaking BCs with Benzoate or Biphenyl

We herein define “soaking” as a strategy to load BCs with carbon/energy sources by reaching sorption equilibrium to allow carbon sources to subsequently desorb and become bioavailable to degrading microorganisms. We hypothesize that soaked BCs can slowly release auxiliary carbon/energy sources for attached cells/biofilms to consume when deployed in situ for bioaugmentation. More sorptive BC materials (e.g., GAC) have a higher capacity to store a supplemental carbon source; however, GAC would also bind carbon sources more tightly. This process affects cell attachment and helps provide bacterial energy needs in situ where carbon supply is limited. To test the effects of soaking on LB400 cell attachment, 0.9 g of GAC or corn biochar (LB) was soaked in K1 medium (27 mL) containing either benzoate (43 mg) or biphenyl (150 mg crystal) until aqueous benzoate or biphenyl concentrations reached equilibrium. Two treatment groups were tested after the soaking BCs. In one treatment, the K1 medium containing residual benzoate or biphenyl was removed and replaced with 27 mL of fresh K1 medium without additional benzoate or biphenyl, such that the only carbon sources were those sorbed onto BCs (“soaked”). In the other treatment, a K1 medium containing residual benzoate or biphenyl was used. Treatments were inoculated with 3 mL of benzoate- or biphenyl-grown LB400 cells and compared to controls where LB400 cells were growing on the same carbon source (43 mg of benzoate or 150 mg of biphenyl) and BC mass (0.9 g of GAC or LB) without soaking. Duplicates were used under all conditions.

### Statistical Analysis

Statistical analysis was conducted in GraphPad Prism 9, R Studio, and Microsoft Excel. Normality of experimental data was tested by Shapiro–Wilk and Kolmogorov–Smirnov tests and normal probability plots. A pairwise two-sided *t* test or one-way ANOVA was applied to test for differences between treatment means depending on the experimental design. Differences were considered significant at 95% confidence level (alpha = 0.05).

## Results and Discussion

### Black Carbon Characteristics Influence Differences in Attached Cell Abundances

**Figure 1:**
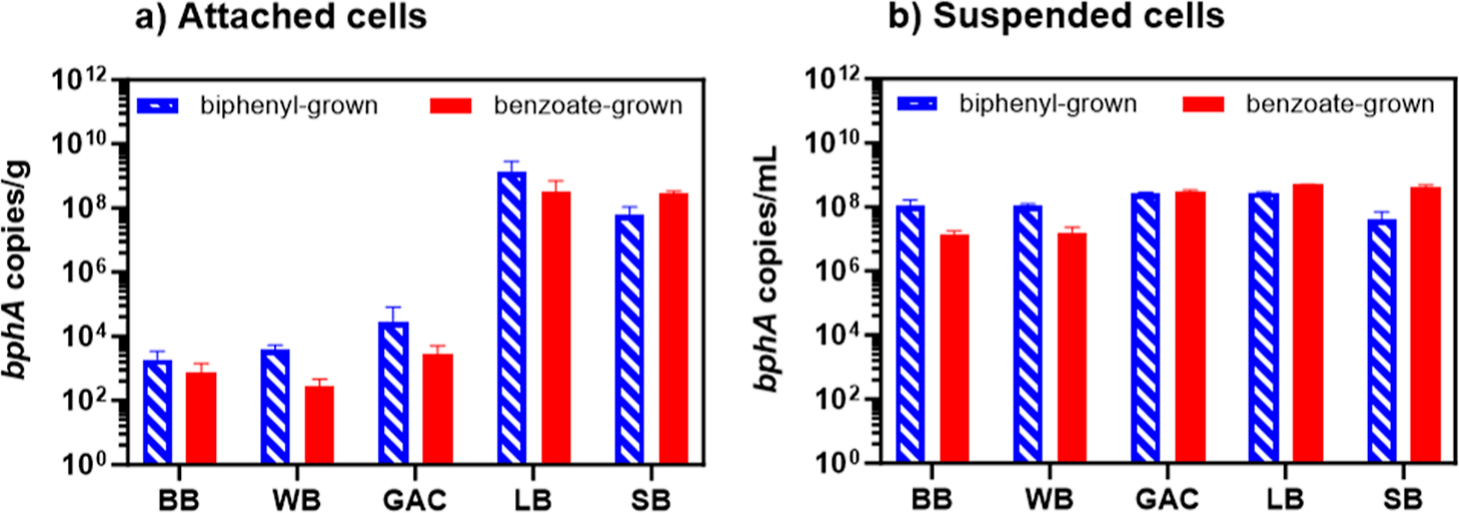
*bphA* abundance on the BC surface (attached) and suspended in the liquid phase after 10 days incubation with (a) attached cells and (b) suspended cells. Either biphenyl or sodium benzoate (equivalent theoretical oxygen demand) was the carbon and energy source. The unit “*bphA* copies/g” in (a) was based on wet weight. LB: large corn kernel biochar, SB: small corn kernel biochar, BB: bamboo biochar, WB: wood biochar, and GAC: granular activated carbon (*n* = 2). The error bars represent the standard deviations of biological duplicates and qPCR technical replicates. Note the log-scale on the *y*-axis.

The abundance of LB400 cells attached to black carbon (BC) surfaces, as *bphA* copies/g, was influenced by different BC materials during growth on either biphenyl or benzoate (Figure 1a). Abundance of *bphA* on each BC type indicated that *bphA* copies/g on small sized corn kernel biochar (SB) or large sized corn kernel biochar (LB) was more than 3 orders of magnitude higher during LB400 growth on biphenyl, and at least 4 orders of magnitude higher during LB400 growth on benzoate, than the other feedstocks we tested (Figure 1a). When pooling all *bphA* copies/g into two groups (Group A: SB and LB corn biochar versus Group B: GAC, BB, and WB), there were no significant differences at the 95% confidence level (*p* = 0.067) between the two groups during growth on biphenyl, while Group A was over 5 orders of magnitude more (*p* = 0.0092) *bphA* copies/g than Group B during growth on benzoate. Pairwise comparisons between *bphA* copies/g on BCs demonstrated significant differences between SB and BB, WB, and GAC during growth on biphenyl (*p* = 0.023) but no significant differences between LB and BB, WB, and GAC (*p* = 0.076), likely due to high variance within LB replicates. Similar *bphA* copies/g differentiation between SB and GAC, WB, and BB and between LB and GAC, WB, and BB occurred during growth on benzoate (*p* = 0.0003 and *p* = 0.18, respectively). There were no significant differences between *bphA* copies/g on GAC, WB, and BB during growth on biphenyl (*p* > 0.06) and during growth on benzoate (*p* > 0.11). The carbon source type (biphenyl or benzoate) significantly affected *bphA* copies/g on SB and WB (*p* < 0.0025), but not on GAC, BB or LB (*p* > 0.15). Cell growth with benzoate yielded ∼5 times higher *bphA* copies/g on SB and ∼14-fold lower *bphA* copies/g on WB compared to biphenyl. The different *bphA* abundances on various BCs indicated the propensity of PCB-degrader LB400 to attach to specific surface materials and its potential activity in bioaugmentation applications.

The abundance of suspended LB400 cells grown on either biphenyl or benzoate, quantified as *bphA* copies/mL, was also impacted by the presence of different BCs (Figure 1b). The highest *bphA* copies/mL in biphenyl-grown cultures were observed when either LB or GAC was present—at least 2-fold more than other BC types (*p* < 0.0003), while the lowest *bphA* copies/mL occurred with SB present (*p* < 0.026). We also observed >2 times higher *bphA* copies/mL in biphenyl-grown cultures with GAC than WB or BB present (*p* < 0.0004). The *bphA* copies/mL in benzoate-grown cultures exhibited significant differences among BCs in the following order: SB or LB > GAC > BB or WB (*p* < 0.00005). Each group started with the same initial quantity of suspended biomass but yielded up to 37-fold differences (LB vs BB) in *bphA* copies/mL among BC feedstocks. The impacts of BC addition are consistent with previous reports of biochar or AC presence altering microbial abundance and diversity. We hypothesize that this phenomenon could be influenced by variable nutrient content (e.g., C, N, P, and S) and physicochemical properties (e.g., pH, electrical conductivity, pore size, and surface area) among the BC feedstocks, which could contribute to the abundance and activity of suspended cells.^41,54,56,82,83^

In biphenyl-grown cultures, there were no significant *bphA* abundance differences between suspended and attached cells when LB was present (*p* > 0.08), but when SB, GAC, WB, and BB were present, *bphA* copies of suspended cells were more than 20-fold higher than *bphA* copies of attached cells (*p* < 0.02). In benzoate-grown cultures, *bphA* copies of suspended cells were at least 38 times greater than *bphA* copies of attached cells (*p* < 0.04) when GAC, BB, SB, and LB were present, while there were no significant differences between suspended and attached cells when WB was present. Another study^41^ similarly reported that certain bacteria, e.g., *Burkholderia* sp., favor planktonic growth while some tend to attach to biochar surfaces. This difference may depend on availability of aqueous growth factors and interactions with biochar, where higher cell attachment to biochar could provide shelter, promote electron transfer, and/or help cells adsorb intermediates to compensate for unfavorable growth conditions.^41^

We hypothesized that sorption potential (*K*_d_), porous character, pH, and surface structure were potential factors driving differences in LB400 cell attachment on the BC materials tested. Corn kernel biochar had a lower *K*_d_, surface area, pore size, and total porous volume than those of GAC, BB, and WB (Table 1). For example, the average benzoate *K*_d_ of corn kernel biochar (0.0014 L/g) was >100 times less than GAC (0.164 L/g). The mesopore size (defined as 2–50 nm) and mesoporous volume of corn kernel biochar (3.23 nm and 0.028 cm^3^/g, respectively) were smaller than those of GAC (3.76 nm and 0.50 cm^3^/g). The corn kernel biochar BET surface area (3 m^2^/g) and total porous volume (5.66 × 10^–3^ cm^3^/g) were also at least an order of magnitude lower than GAC (surface area: 386 m^2^/g; porous volume: 0.482 cm^3^/g). This indicates that BCs with lower *K*_d_, surface area, pore size, and porous volume potentially provide environments beneficial to cell attachment, which is consistent with reports that cell attachment increased with the decreasing pore size.^84,85^ Lower sorption capacity makes the aqueous carbon source more bioavailable to LB400 cells growing with corn kernel biochar than other BCs tested. In contrast, strong BC sorption behavior hampers LB400 attachment, consistent with previous observations with GAC,^54,56−58^ and even potentially affects carbon source bioavailability to the indigenous microbial community.^56,58^

BC pH effects likely impacted the LB400 cell attachment. The initial pH values of solutions when BC was placed in DI water (pH = 5.89) were: 5.40 (GAC), 7.88 (corn kernel), 9.58 (wood), and 10.11 (bamboo). After adding DI-rinsed BCs in buffered K1 medium (neutral pH) for cell attachment, we found that BC with circumneutral pH had the highest *bphA* copies/g, while more basic or acidic BCs were less beneficial to LB400 cell attachment (at least 3 orders of magnitude). These results are consistent with observations that pH buffering capacity contributed by BC alkalinity correlated with microbial survival.^40,86^ Here, GAC exhibited lower alkalinity than corn kernel biochar while the wood and bamboo biochars exhibited the opposite, which was potentially related to pyrolysis temperature and surface functional groups.^40^ GAC, wood, and bamboo biochars, however, may be less suitable than corn kernel biochar for LB400 survival and attachment. Even if BCs were prewashed with buffer to remove impurities (herein defined as pyrolysis residual ash), we would not expect fundamental BC properties (e.g., carboxyl, hydroxyl, phenolic surface functional groups)^53,87^ to be altered with rinsing.

BC surface characteristics may affect cell attachment differently. In our study, the coarser GAC surface contrasted with flatter biochar surfaces (Figure S9), which—although initially somewhat counterintuitive as GAC surface roughness provides more surface area for cell attachment,^84^—aligns with reports that smooth and flat biochar surfaces more easily accommodated bacteria than AC surfaces.^54,88,89^ We found among three relatively smooth and flat biochars that corn kernel biochar displayed the highest LB400 attachment, indicating that surface structure potentially plays a secondary role in influencing cell attachment compared with features such as sorption potential, porous character, and pH.^54^ Our experiments could not explicitly identify key factors influencing cell attachment; however, postanalysis indicated that mesopore size may be an important parameter (Table S4, *p* = 0.0833, rho = −0.9) compared to others measured (*p* > 0.27, rho: −0.5 to −0.7) at the 90% confidence interval. This phenomenon was consistent with prior studies wherein pore size influenced bacterial attachment and reactivity of BCs.^53,54,57,90^ Because corn kernel biochar has greater potential to host elevated attached LB400 cell abundances, this fundamental characterization is likely to be important in bioaugmentation applications aimed at achieving sustained PCB biodegradation in sediments.

### LB400 Cells Attached to BC Are Viable and More Active for Contaminant Biodegradation than Suspended Cells

**Figure 2:**
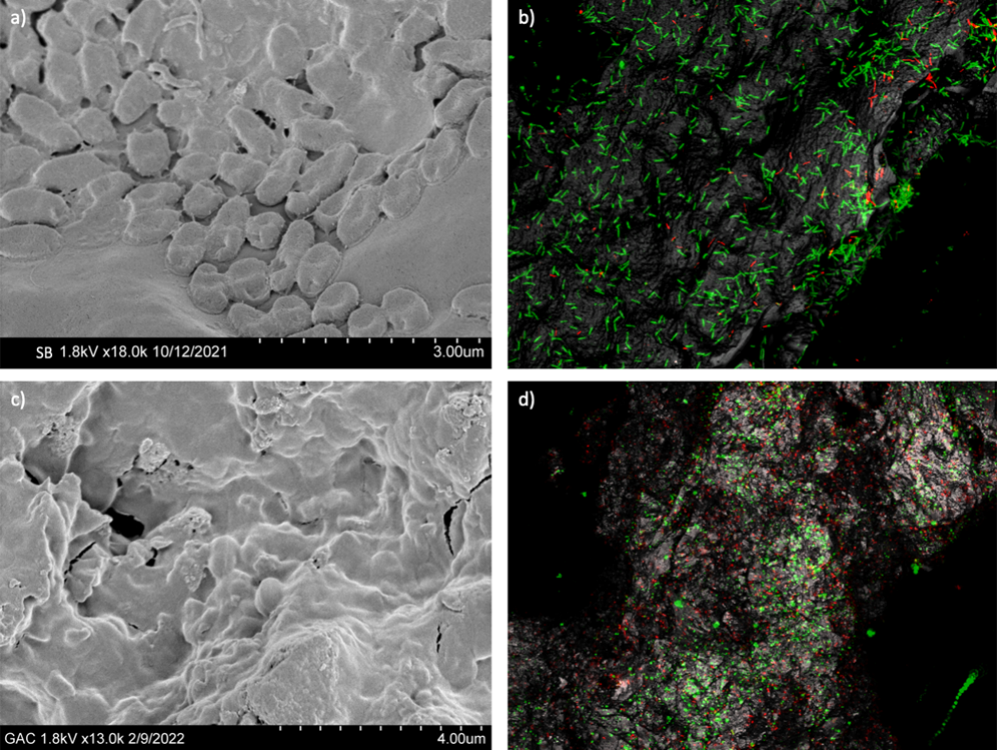
(a) SEM imaging of LB400 cells attached to a SB surface; (b) CLSM imaging of live and dead LB400 cells on SB; (c) SEM imaging of LB400 cells attached to a GAC surface; (d) CLSM imaging of live and dead cells on GAC. Red dots in CLSM imaging represent dead cells, and green dots represent live cells. All cells shown were grown with biphenyl as sole carbon and energy source supplied.

Using both SEM and CLSM imaging, we observed LB400 cells attached to the SB and GAC surfaces (Figure 2). Corn kernel biochar was chosen because of its highest surface *bphA* abundance, while GAC was used as representative “baseline” BC because it has been previously applied with functional microbes for PCB bioremediation.^38,49^ CLSM images also illustrate the distribution of live and dead LB400 cells. Using partial surface CLSM images (Figure 2b,d), we observed a higher live-to-dead cell ratio on SB (4.28) than on GAC (1.43) (Table S5). Although total cell numbers on GAC surfaces were higher than on SB surfaces in these images, overall 44.7% of the cells on GAC surfaces were dead, while only 15.8% of cells were dead on SB surfaces. This is consistent with our hypothesis that GAC characteristics (higher sorption capacity, lower alkalinity, and more acidic surfaces) pose environmental stress to LB400 cells compared to corn kernel biochar (low sorption capacity, moderate alkalinity, and neutral surface).

In addition, the average distance from cells to the SB surface (4.74 μm) was larger than that to the GAC surface (1.46 μm), while the distance between dead cells to surfaces tended to be shorter than live cells (1.63 μm to SB and 0.69 μm to GAC) (Table S5). This implies that LB400 cells attached to SB surfaces were denser than on the GAC surface; an outer layer of cells creates a beneficial environment for cell growth such as higher oxygen level and carbon sources. Denser cell attachment on SB could imply lower oxygen transfer and more cell death compared to cells attached to GAC,^91^ which is opposite to our observations. Nevertheless, attached cells were scattered on BC surfaces instead of continuously overlapped (Figure 2b,d), implying that the oxygen transport in the biofilm may not be limited. Alternatively, a prior study^92^ reported that oxygen deficiency induced biofilm formation, thus potentially creating a positive feedback on cell attachment and cell survival. Complete characterization of cells attached to BC surfaces is difficult because CLSM can only visualize small areas with high resolution; thus, multiple regions on different BC pieces were evaluated and demonstrated consistent results.

**Figure 3:**
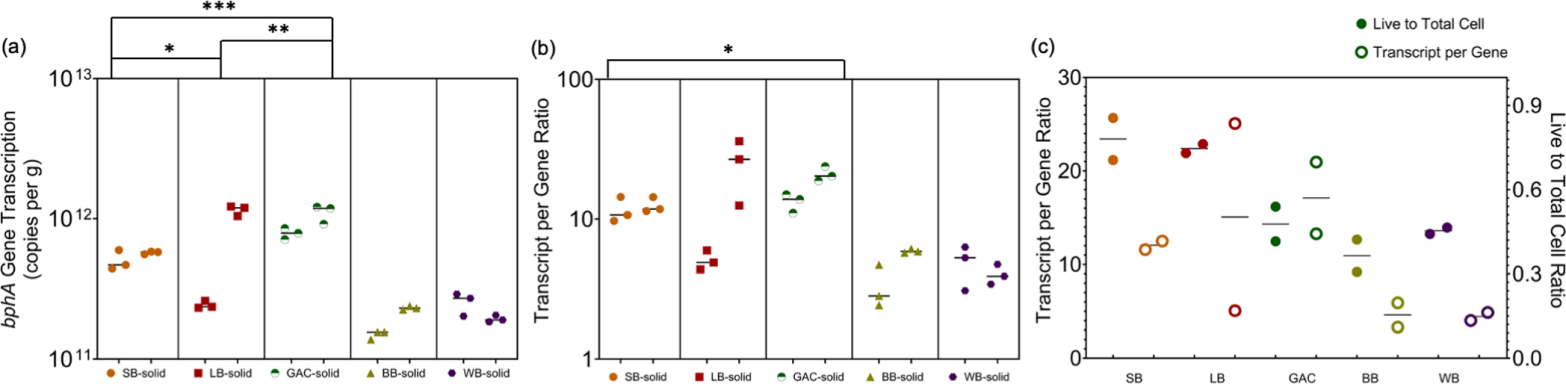
(a) *bphA* transcription (*bphA* transcripts per gram of BC) differences among five BCs. (b) Gene expression level (transcript per gene ratio) differences among LB400 cells attached to different BCs. (c) Comparison between transcript per gene ratios and live to total cell ratios of LB400 cells attached to BC. Significance labels: “*” *p* < 0.05, “**” *p* < 0.01, and “***” *p* < 0.001. Both BB and WB exhibited significantly lower (*p* < 0.01) *bphA* transcription and transcript per gene ratio than the other three BCs, although “*” was not labeled in figure. The unit “copies per gram” in (a) was based on wet weight. For each treatment group in (a) and (b), biological duplicates are displayed as two separate data point clusters while each cluster represents technical triplicate qPCR measurements. Horizontal bars represent the median of the technical replicates. For (c), data points represent biological replicates, and horizontal bars represent the median. LB: large corn kernel biochar, SB: small corn kernel biochar, BB: bamboo biochar, WB: wood biochar, and GAC: granular activated carbon.

*bphA* gene expression of both suspended and attached biphenyl-grown LB400 cells after 10 days of incubation was quantified by RT-qPCR to compare the activity of cells attached to the four different BC materials. This study is the first to demonstrate that *bphA* expression in attached LB400 cells was significantly influenced by the BC materials. *bphA* transcript abundance, which ranged from 1.38 × 10^11^ to 1.22 × 10^12^ transcripts/g, was highest on GAC followed by LB, SB, and BB/WB (*p* < 0.02) (Figure 3a). When normalized to *bphA* copies/g, the resulting transcript-per-gene ratio varied from 3.3 to 25 with no significant differences (*p* > 0.05) between LB and SB or LB and GAC (Figure 3b). This implies that certain BC characteristics, shared by GAC and LB/SB but not WB and BB, were beneficial to *bphA* expression in the attached cells. Interestingly, LB, SB and GAC harbored at least 3-fold more live attached cells than other biochars (WB and BB) on average.

Comparing the live to total cell ratios to the transcript/gene ratios indicated that, in general, ∼a 2-fold higher live cell fraction on BC surfaces yields >3-fold higher *bphA* expression levels except for GAC (Figure 3c). Although GAC hampers carbon source bioavailability due to greater sorption, the number of live cells attached to the surface expressing *bphA* was also elevated. The exact mechanism driving gene expression changes in attached cells resulting from different BC surfaces is poorly understood, but this novel finding implies that BC materials can be tuned to regulate microbial activity and achieve a desirable biodegradation performance. Furthermore, by combining CLSM with RT-qPCR to track cell distribution and activity on BC surfaces and pore sites,^93^ we could obtain more effective evidence of biodegradation potential and even study the fate of microorganisms after field deployment.

It is notable that the transcript per gene ratio was unity for suspended LB400 cultures while the ratios for attached cells were >10 (Figure S10). Expression of *bphA* in attached LB400 cells was significantly greater (*p* < 0.0001), ranging from 7-fold to 18-fold, than in suspended cells after 10 days growth on biphenyl. Both transcript per gene ratio and transcript abundance metrics emphasize the relative differences in LB400 activity between suspended and attached phases. A *bphA* transcript per gene ratio of 1 indicates that after 10 days growth on biphenyl, *bphA* was induced at a relatively low level, which is consistent with previous work.^94^ In attached LB400 cells, biphenyl induced *bphA* expression at higher levels, regardless of the BC materials used. Differences in *bphA* expression levels between suspended and attached cells may have been influenced by variability in cell growth status or interactions with BC materials. After 10 days of growth on biphenyl, suspended LB400 cells entered the stationary phase under the conditions of our experiment (Figure S11). Nevertheless, attached LB400 cells maintained elevated expression levels compared to suspended cells after 10 days. The elevated *bphA* expression level in BC-attached cells compared to that in suspended cells demonstrated that BC addition influences LB400 activity and LC-PCB biodegradation potential.

### RNA-seq Reveals Differences in Gene Expression between Attached and Suspended Cells and among Different BC Feedstock Surfaces

**Figure 4:**
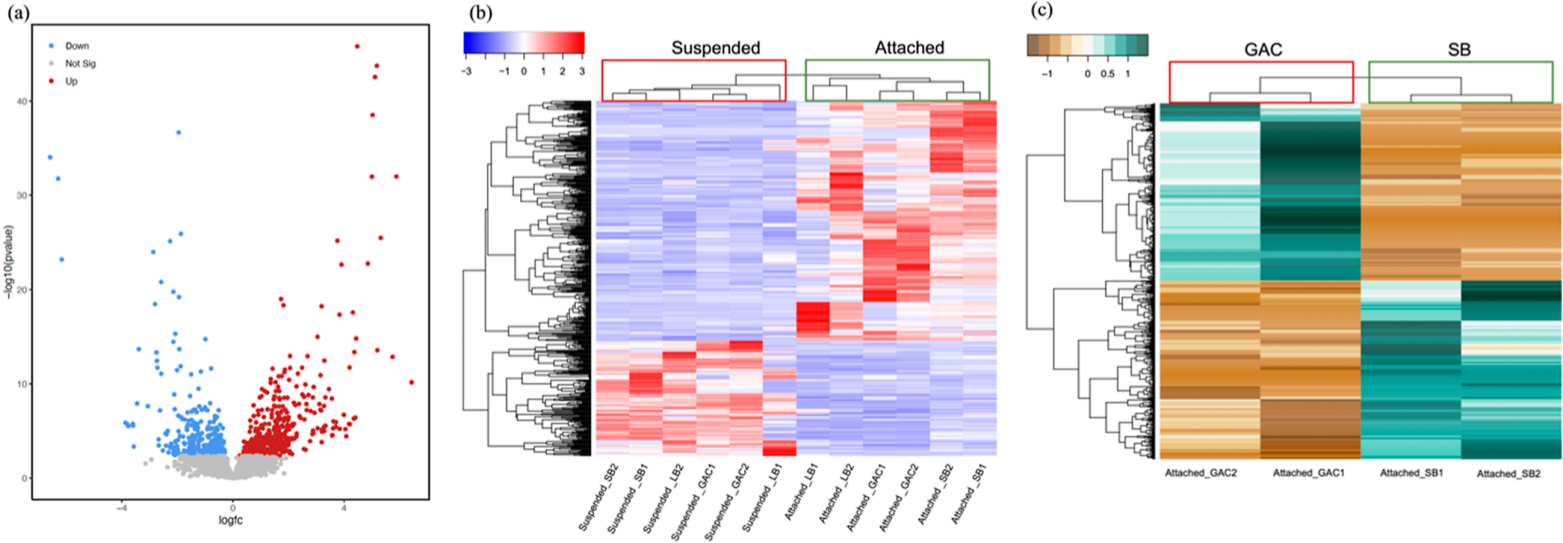
(a) Volcano plot representation of differential gene expression between attached and suspended phase of LB400 growing in the presence of BC. Red dots represent upregulated genes (log_2_(FC) > 0, adjusted *p* < 0.05) in attached cells compared to suspended cells, blue dots represent downregulated genes (log_2_(FC) < 0, adjusted *p* < 0.05) in attached cells compared to suspended cells, and gray dots represent genes without significant differences between attached and suspended cells. (b) Hierarchical cluster analysis (HCA) heatmap of differential gene expression between attached and suspended LB400 cell growing in the presence of BC. Red bars represent upregulated genes while blue bars represent downregulated genes. The HCA also reveals important differences in gene expression between suspended and attached cells. (c) Differential gene expression HCA of LB400 cells attached to either SB or GAC. Green bars represent upregulated genes while yellow bars represent downregulated genes. Both heatmaps were plotted by using Z-scores, standardized based on row, and describe the significant gene expression level of selected genes passing the threshold (log_2_(FC) > |±1|, adjusted *p* < 0.05). The Z-score is the number of standard-deviations that a value was away from the mean of all the values in the same group. Hierarchical clustering was based on the Euclidean distance of each row and demonstrated the similarity of different genes. SB: small corn biochar, LB: large corn biochar, and GAC: granular activated carbon. Branches on the left depicted the cluster of selected genes and branches on the top demonstrated the cluster of samples.

Differential gene expression (DE) analysis indicated notable differences between attached and suspended LB400 cells. There were 744 differentially expressed genes (*p*-adj < 0.05) in total, 450 of 744 were upregulated in attached cells compared to suspended cells and 21 of 450 genes were highly expressed (log_2_(FC) > 4, *p*-adj < 0.02) (Figure 4a). Differently expressed genes were located throughout the LB400 genome (Figure S12).^95^ Hierarchical clustering analysis demonstrated that DE in suspended groups was distinct and separate from that in the attached groups (Figure 4b). Among the differentially expressed genes passing the threshold (log_2_(FC) > |±1|, *p*-adj < 0.05), approximately two-thirds were upregulated in attached cells compared to suspended cells, while another one-third were downregulated (Figure 4b). In concurrence with our RT-qPCR findings, *bphA* was significantly more expressed (log_2_(FC) > 1.5, *p*-adj < 0.001) in attached cells than in suspended cells along with genes that comprise the entire *bph* pathway that converts biphenyl to benzoate (log_2_(FC) > 1.35, *p*-adj < 0.001) (Table S6). Furthermore, the *box* pathway, which catalyzes metabolism of benzoate to 6-hydroxy-3-hexenoyl-CoA,^67^ was highly expressed in attached cells compared to suspended cells (log_2_(FC) > 4, *p*-adj < 0.001) (Table S7). Expression of *box* in biphenyl-grown LB400 cells is expected as benzoate is a *bph* pathway product.^67^ This finding emphasized that *bphA* was not incidentally expressed, but rather the entire PCB biodegradation pathway was upregulated by the addition of BCs.

LB400 cells also displayed DE when attached on different BC surfaces (Figures 4c, S13 and S14). There was an average of 177 upregulated genes in SB-attached cells compared to 82 in LB-attached cells. Furthermore, an average of 499 upregulated genes in GAC-attached cells compared to 504 in SB-attached cells and an average of 472 upregulated genes in GAC-attached cells compared to 332 in LB-attached cells. Variations in the number of upregulated genes between SB and LB may be related to their size differences and the effects of grinding on surface structure. This observation supports our novel findings that BC feedstocks significantly influenced the gene expression of attached LB400 cells. Although *bph* pathway gene expression was not significantly different between corn kernel biochar and GAC (*p* > 0.11), the box pathway was upregulated in SB-attached cells compared to GAC-attached cells (log_2_(FC) > 0.93, *p*-adj < 0.01) during growth on biphenyl but not benzoate, indicating that biphenyl was degraded into benzoate.

Interestingly, we also found that exopolysaccharide (EPS) production and transport pathways (EPS pathway I) were upregulated in LB-attached cells compared to GAC-attached cells (log_2_(FC): 4.3–7.5; *p*-adj < 0.05; Figure S15, Table S8). EPS biosynthesis involves EPS precursors (e.g., UDP-glucose and UDP-glucuronic acid) and EPS export across cell membranes.^96,97^ Another EPS production pathway (EPS pathway II) was also upregulated in both SB- and LB-attached cells compared to GAC-attached cells (log_2_(FC): 1.0–2.9; *p*-adj < 0.01; Table S9). These findings provide evidence that LB400 cells were not simply attached to but also formed biofilms on both sizes of corn kernel biochar, further emphasizing the potential importance of biochar type to drive microbial function (e.g., improving LC-PCBs biodegradation potential) beyond functioning solely as a cell carrier. Although we cannot provide a conclusive mechanistic explanation based on these data, we hypothesize that our observed phenomenon could be related to interactive forces between cells and the solid surface. More specifically, surface physicochemical properties of both cells and solid surfaces could affect adhesion tendency, including BC characteristics discovered important in this study (e.g., sorption capacity, pore size, pH, and surface structure), as well as hydrophobicity of solid surface and cells, surface potential, and hydrogen bonding energy.^98−100^ Microorganisms inhabiting biofilms secrete EPS that capture and concentrate nutrients, increasing resistance to exogenous stressors, creating a protective environment to improve cell longevity, and allowing enhanced gene expression levels compared to planktonic cells.^38,42^ To the best of our knowledge, aerobic PCB-degrading biofilm formation on biochar has not been previously demonstrated.^46,57^

A pathway involved in autoinducer AI-1, *N*-acylhomoserine lactone (AHL) biosynthesis and fatty acid degradation was upregulated in SB-attached compared to GAC-attached cells (log_2_ (FC) > 2.4, *p*-adj < 0.02; Table S10). AHLs, generated from the reaction of acyl-(acyl-carrier protein) and *S*-adenosyl-methionine (Figure S16), are common signal molecules that regulate quorum sensing (QS) among cells.^101−104^ The elevated expression of EPS and AHL biosynthesis genes in SB/LB-attached cells compared with GAC-attached cells is further evidence of biofilm formation on corn kernel biochar. EPS production is positively regulated by a BraIR-like QS in LB400,^102^ and we are the first to report that LB400 on corn kernel biochar excreting EPS was potentially controlled by the *xenI2* and *xenR2* system. Upregulation of AHL biosynthesis in SB-attached cells than GAC-attached cells was reasonable as adsorption could inhibit QS.^105^

### Biodegradation Potential of LB400 Cells Attached to BCs

We investigated the ability of BC-attached LB400 cells to degrade chemicals (e.g., benzoate and acetate). LB400 cells attached to SB and GAC fully degraded 6 mM benzoate within 3 days (Figure S17) and 4 mM acetate within 1 day (Figure S18). Acetate, a substrate readily metabolized via the TCA cycle, was consumed as expected by cells attached to both BCs with approximately 11% sorption, as measured by sterile BC controls (Figure S18). Acetate utilization is a first indication of the viability and activity of attached LB400 cells. We chose benzoate as a model chemical to test the activity of attached LB400 cells because benzoate, a *bph* pathway inducer and product, is less sorptive (Table 1), more soluble, and thus more easily measured in the aqueous phase than biphenyl or LC-PCBs. We did not incorporate PCBs in these experiments to avoid competing BC sorption processes and the complexity of PCB dissolution and extraction, thus allowing specific study of BC impacts on biofilms; future work will incorporate PCBs. Both sorption and biodegradation mechanisms can contribute to the observed loss of benzoate in experiments with LB400 cells attached to SB or GAC. Because SB sorbs benzoate less than GAC (Table 1), benzoate biodegradation contributed more than sorption to benzoate loss in the SB treatment. Benzoate sorbed to GAC immediately after addition, which led to a faster apparent loss of benzoate in the GAC treatment. Using multiple lines of evidence to investigate the biodegradation potential of LB400 cells attached to BC materials is important to help predict their performance in PCB-contaminated sediment applications.

### Soaking Sorptive BC Materials with Benzoate Increased Abundance of Attached LB400 Cells

**Figure 5:**
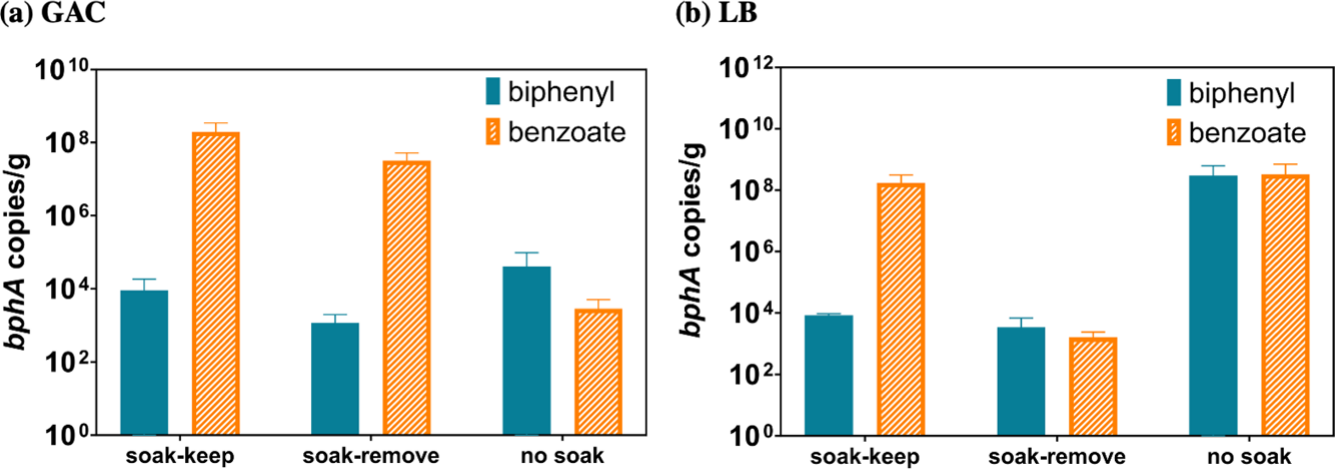
*bphA* abundance on GAC or LB surface with either 43 mg of benzoate or 150 mg of biphenyl after 10 days incubation. Although the highest *bphA* abundance in “no soak” was over 10^8^ copies per gram of LB and much higher than “soak-remove”, due to large biological differences within “no soak”, there was no significant differences (*p* > 0.05) between “no soak” and “soak-remove” or between “no soak” and “soak-keep”. In “soak-keep”, K1 medium containing residual benzoate or biphenyl was used. In “soak-remove”, the K1 medium containing residual benzoate or biphenyl was removed and replaced with fresh K1 medium without additional carbon sources. “No soak” represents control groups. The unit “*bphA* copies/g” was based on wet weight. LB: large corn biochar; GAC: granular activated carbon. All of the controls and treatments were conducted in duplicate. Error bars represent the standard deviations of biological duplicates.

Because of the large sorption capacity differences between GAC and LB, we investigated the impacts of “soaking” BCs with carbon sources on carbon source bioavailability and subsequent attachment of LB400 to these BCs. “Soaking” is a strategy for loading carbon/energy sources onto BC to extend cell activity. Compared to “no soak” treatments, soaking GAC (soak-keep and soak-remove) with benzoate increased *bphA* copies/g by over 4 orders of magnitude (*p* < 0.007), while soaking GAC (soak-keep and soak-remove) with biphenyl showed no significant differences in *bphA* copies/g among all treatments and controls (*p* > 0.25, one-way ANOVA) (Figure 5). Based on *K*_d_ estimates (Table 1), we expected that benzoate distribution and bioavailability would impact cell attachment more than biphenyl. Soaking the less sorptive LB with either biphenyl or benzoate (soak-keep and soak-remove) showed no significant differences in *bphA* copies/g compared with “no soak” treatments (biphenyl: *p* > 0.08, one-way ANOVA; benzoate: *p* > 0.18, two-sided *t*-test). Removing residual benzoate after soaking (soak-remove) yielded a decrease in *bphA* copies/g by >4 orders of magnitude compared to maintaining residual benzoate after soaking (soak-keep; *p* = 0.03) because there was less benzoate sorption to LB than GAC (Table 1). Soaking with benzoate (soak-keep) significantly decreased *bphA* copies/mL for both BCs (*p* < 0.003) at least 1.3 times compared to no-soak controls (Figure S19). Removing the residual carbon source after soaking but prior to adding LB400 (soak-remove) significantly decreased the resulting *bphA* copies/mL by >18-fold (*p* < 0.003) on average in LB than no-soak, but the phenomenon was less pronounced in GAC (i.e., only 3-fold difference). This demonstrated that GAC desorbed more benzoate than LB to support cell growth after soaking. Interestingly, cell attachment on GAC was lower than on LB due to high sorption capacity and concomitant decreased carbon source bioavailability, but GAC could improve cell attachment by compensating bioavailability via desorption. Both sorption and desorption affected carbon source bioavailability and biodegradation efficiency. Specifically, the degree of desorption can predict carbon source bioavailability in a biochar-bacteria system, where desorption was limited at low carbon source concentrations but promoted at high adsorption.^106^ Thus, BC soaking strategies may improve cell attachment and provide continuous carbon source desorption to bacteria for in situ application when bioaugmenting with more sorptive materials such as GAC.

## Environmental Implications

Our study is the first report that BC feedstocks significantly influenced microbial gene and pathway expression and provides a new understanding of interactions between microbes and BC surfaces. Different BCs are critical drivers that regulate microbial activity, attached cell abundance, and biofilm formation. Desirable, and potentially tunable, BC feedstocks can be valuable microbe carriers for bioremediation processes, wherein bacteria attached on BC could secrete EPS to maintain more stable cell numbers and resist environmental changes,^38,40,41^ thus yielding higher bioactivity. Prior studies that used specific BCs (i.e., GAC)^46−48^ to deliver cells focused more on contaminant removal than cell attachment while having bioavailability limitations.^50,51^ Relatively few studies considered the diversity of BCs.^52^ Our study probed the role of BC sorptive bioavailability on attached cell gene expression and microbial activity. Low sorption capacity BCs beneficially maintained added benzoate with sufficient bioavailability for LB400 without overdosing while sorption helped shorten the mass transfer distance between cells and contaminants.^38^ Understanding the potential drivers of cell-BC interactions and revealing cell–cell communication pathway expression add to knowledge concerning microbial fate on BC and strategies for maintaining cell activity, which could be critical in optimizing bioaugmentation strategies. Although biodegradation of PCBs was not directly measured in this study, complementary molecular tools with CLSM cell imaging helped us understand microbe–BC interaction complexities and provided multiple lines of evidence for the biodegradation potential.

The activity differences between attached and suspended LB400 cells highlight the advantages of bioaugmentation with BC-attached cells and hold implications for technology scale-up. Assessing whether BC addition specifically facilitates or inhibits LC-PCB biodegradation was beyond the scope of this study, which is environmentally relevant but largely influenced by bioavailability and competing sorption of BCs. Instead, we focused on evaluating the effects of BC feedstock and surface properties on the biodegradation potential through the assessment of gene and pathway expression and biofilm formation. Upregulated *bph* and *box* pathway gene expression in attached cells and on certain BCs further indicate improved LC-PCBs biodegradation potential. In general, LC-PCBs biodegradation activity and potential could be tuned by choosing appropriate BC feedstocks and/or adjusting BC attributes to enhance the persistence of bioaugmented microbes and resultant efficiency of LC-PCB removal.^50,54,56−58^ It is important to note that in real environments the addition of BCs would complicate the mass transfer and distribution of PCBs by adding a sorbed phase distribution.^17,56^ In addition, external environmental stresses such as low oxygen level, high salinity effects of marine estuaries, and competition with indigenous microorganisms were not considered in the study but might potentially affect the activity of BC-attached LB400 when applied on-site.^48,107−109^ One approach to help mitigate these environmental factors could be encapsulation of BC-coated biofilms, providing mechanical protection and sustainable active functional microbes.^110,111^ Overall, developing desirable cell attachment on BC is a sustainable alternative approach compared to dredging with both economic benefits and lessened environmental disruption.^48,107−109^

To the best of our knowledge, this is the first study to report the importance of specific biochar on aerobic PCB-degrading cell attachment and biofilm formation on BC and investigate key BC factors that drive LB400 cell attachment, biofilm formation potential, and biodegradation pathway expression, with implications for improving LC-PCBs biodegradation performance. Notably, we discovered that different BCs influence gene expression levels in LB400, emphasizing the importance of biomass carriers in aerobic PCB degradation. This work demonstrates that a diverse suite of BCs (i.e., biochars of varied feedstocks, not only GAC) holds potential to deliver abundant and active functional microorganisms for LC-PCB bioremediation in sediment. LC-PCBs are the most volatile PCBs and cause direct exposure to humans through inhalation, but have received comparatively less attention than HC-PCBs.^112^ This research provides potential biological approaches to break the sediment-to-air LC-PCB human exposure pathway and decrease the total contaminant mass in sediment, thereby benefiting public health and ecosystems.

## Data Availability

Read count tables and DeSeq2 differential expression data are available under GEO Accession number GSE246487. Raw transcriptomic reads are available under BioProject number PRJNA1033190.

## Supporting Information Available

Supporting experimental methods/results for experimental design, medium preparation and carbon source addition; cell attachment visualization; modified DNA extraction procedures; DNA and RNA yields (Table S1); qPCR analysis and QA/QC (Tables S2 and S3; Figures S1–S6); biphenyl and benzoate sorption (Figure S7); biphenyl, benzoate, and acetate analysis (Figure S8); Black carbon characterization (Figure S9); Correlations between BC properties and *bphA* abundance in attached cells (Table S4); Viability and activity of attached LB400 cells (Table S5, Figures S10 and S11); RNA-seq of attached and suspended LB400 cells (Figures S12–S16, Tables S6–S10); Chemical biodegradation by BC-attached LB400 (Figures S17 and S18); Soaking BC with biphenyl or benzoate (Figure S19) (PDF)
