# Supplementary material for: Black Carbon Impacts on Paraburkholderia xenovorans Strain LB400 Cell Enrichment and Activity: Implications toward Lower-Chlorinated Polychlorinated Biphenyls Biodegradation Potential
Source: Environ Sci Technol. 2024 Feb 15;58(8):3895–907. doi: 10.1021/acs.est.3c09183 (PMC10902836; doi:10.1021/acs.est.3c09183)
Supplement: Supplementary file 1 — es3c09183_si_001.pdf [file es3c09183_si_001.pdf]

Supporting Information

Black Carbon Impacts on *Paraburkholderia xenovorans* Strain LB400 Cell Enrichment and Activity: Implications toward Lower-Chlorinated Polychlorinated Biphenyls Biodegradation Potential

Qin Dong,[†,‡] *Gregory H. LeFevre*[†,‡] and *Timothy M. Mattes*[†,‡,*]

[†] Department of Civil and Environmental Engineering, University of Iowa, 4105 Seamans Center, Iowa City, Iowa, 52242, United States

[‡] IIHR—Hydroscience and Engineering, University of Iowa, 100 C. Maxwell Stanley Hydraulics Laboratory, Iowa City, Iowa, 52242, United States

[*]**Corresponding Author**

Email: tim-mattes@uiowa.edu
Phone: 319-335-5065
Department of Civil and Environmental Engineering
4105 Seamans Center for Engineering, University of Iowa,
Iowa City IA, 52242, United States

This supplemental information document includes:

Supplementary methods (Section S1.1-S1.6), Supplementary results (Section S2.1-S2.4), Figures S1-S19, Tables S1-S10

S1. Supplemental Methods and Materials

S1.1 K1 Medium Preparation and Carbon Source Addition

K1 medium was made by adding 100 mL K1 10× stock solution and 20 mL Hutner mix into 880 mL sterile deionized (DI) water. K1 10× stock solution was made by adding 15.9 g K_2HPO_4, 2.5 g $(NH_4)_2SO_4$, and 1.77 g $NaH_2PO_4{\cdot}H_2O$ into 1000 mL DI water. After autoclaving K1 10× stock solution for 30 minutes at 121˚C, sterile vitamins were added, including 250 μL 10 mg/mL of thiamine, nicotinic acid, nyo-inositol, and riboflavin in phosphate buffer and 25 μL 2 mg/mL of D-biotin in methanol. Hutner mix contains various salts and "Metals 44" solution as described below. First, nitrilotriacetic acid (NTA) (10 g) was added into 600 mL DI water with a magnetic stir bar on a stir plate. The cloudy NTA solution dissolved after adding KOH pellets. Afterwards, 14.5 g $MgSO_4{\cdot}7H_2O$, 3.33 g $CaNO_3$, 9.25 mg $(NH_4)_6Mo_7O_{24}{\cdot}24H_2O$, 99 mg $FeSO_4{\cdot}7H_2O$, and 50 mL Metals 44 were added sequentially. After all chemicals were completely mixed, an additional 400 mL DI water was added into the solution, followed by pH adjustment to 6.6-6.8. Solution was filtered through 0.2 μm filters and stored at room temperature. Metals 44 was made by adding the following chemicals to 400 mL DI water: 1 g EDTA, 4.4 g $ZnSO_4{\cdot}7H_2O$, 2 g $FeSO_4{\cdot}7H_2O$, 620 mg $MnSO_4{\cdot}H_2O$, 119 mg $CuSO_4{\cdot}5H_2O$, 99.4 mg $Co(NO_3)_2{\cdot}6H_2O$, and 70.8 mg $Na_2B_4O_7{\cdot}10H_2O$. A few drops of H_2SO_4 were added to dissolve metals, and the solution was filtered through 0.2 μm filters and stored at room temperature.

Additional carbon sources were added to the K1 medium for cell growth. Based on their theoretical oxygen demand, 5 mM biphenyl crystal or 10 mM sodium benzoate was needed. Depending on the final volume of K1 medium, different mass of carbon sources were added. For example, when the volume of K1 medium is 250 mL, 0.19 g biphenyl crystal or 0.36 g sodium benzoate is needed.

The abundance of bphA in attached and suspended LB400 cells after different incubation periods. The impacts of incubation time on *bphA* abundance in attached cells and suspended cells were tested. The *bphA* abundance of attached cells was lower after a 20-day incubation than after a 10-day incubation (Figure S1a). The *bphA* abundance of suspended cells (Figure S1b, c) was not significantly different ($p>0.05$) after cultures entered stationary phase (~6d, Figure S11).

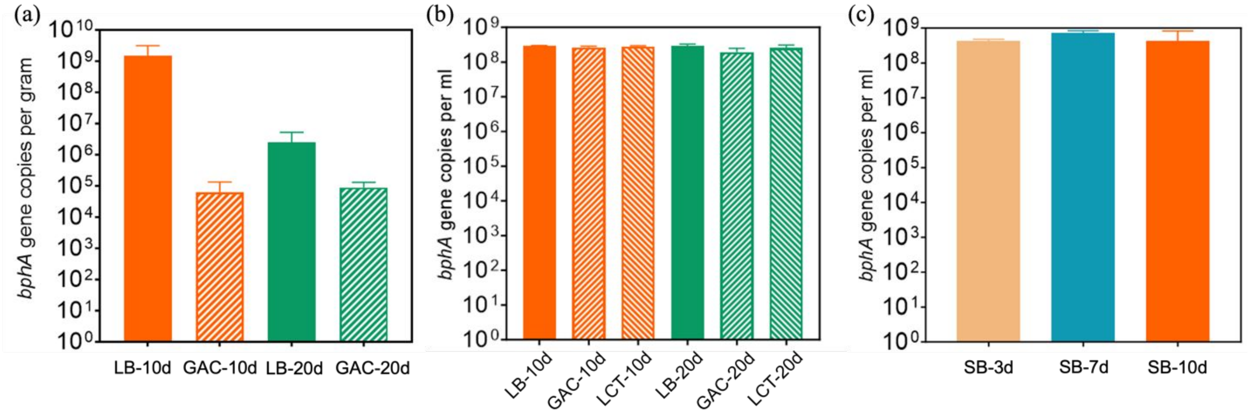

Figure S1. The *bphA* abundance in suspended and attached LB400 cells when 22.8 mg of biphenyl was added as sole carbon and energy source. (a) *BphA* abundance on the BC surface (attached) after 10-day and 20-day incubations. (b) *BphA* abundance in suspended cells after 10-day and 20-day incubation. (c) *BphA* abundance in suspended cells after 3-day, 7-day, and 10-day incubation. The unit in (a) was based on wet weight. LB: large corn kernel biochar, SB: small corn kernel biochar, GAC: granular activated carbon, and LCT: live cell treatment without BC addition. The error bars represent the standard deviation of biological duplicates and qPCR technical replicates. Note the log-scale on the vertical axis.

S1.2 Cell Attachment Visualization

Confocal Laser Scanning Microscopy (CLSM). Cell attached to BC surfaces were stained with 10 μM SYTO9 and 60 μM propidium iodide (PI) in phosphate-buffered saline (PBS) for 15 minutes at room temperature in the dark. After rinsing residual dyes with 1 mL PBS, samples were covered in 90% glycerol with 10 mM Tris-HCl (pH 8) prior to CLSM imaging. Reflectance and fluorescence imaging were observed under 488 nm and 499-600 nm, respectively. PBS was made by adding 8 g NaCl, 0.2 g KCl, 1.44 g Na_2HPO_4, and 0.24 g KH_2PO_4 into 1 L DI water, which pH was adjusted to 7.4.

Scanning Electron Microscopy (SEM). Before inserting samples into SEM chamber, all samples were air dried, coated with silver solution (60% Ag in 1-methoxy-2-propanol, Electron Microscopy Sciences, Hatfield, PA) on an aluminum holder, and mounted with a conductive layer of gold or iridium sputter (EMS 150T ES, Quorum Technologies, Lewes, United Kingdom).

S1.3 Modified Procedures for DNA Extraction

Instead of using a Sterivex filter unit, two mL of liquid samples (diluted as appropriate) were directly added to a glass PowerBead tube with 0.9 mL warm MBL solution and then incubated at 90°C for 5 mins. After cooling for 2 mins, the solution was vortexed at maximum speed for 5 mins and centrifuged at 4000×g for 1 min. The remaining procedures followed the kit.

Table S1. DNA and RNA yields from suspended and attached cells with various BC additions. SB: small corn biochar, LB: large corn biochar, GAC: granular activated carbon, BB: bamboo biochar, and WB: wood biochar. "1" and "2" represent the biological duplicates.

Sample	liquid		solid	
	DNA (ng/μL)	RNA after DNA digestion (ng/μL)	DNA (ng/μL)	RNA after DNA digestion (ng/μL)
SB1	574.00	242.90	40.40	116.10
SB2	370.00	128.40	30.50	117.60
GAC1	165.00	70.30	34.70	122.00
GAC2	258.00	50.50	43.40	110.40
LB1	360.00	108.3	34.00	108.8
LB2	214.00	36.1	43.50	165.3
BB1	-	-	44.20	191.30
BB2	-	-	47.20	151.60
WB1	-	-	33.80	-
WB2	-	-	55.00	-

S1.4 qPCR Analysis and QA/QC

Table S2. Primer sets used in qPCR analysis.

Target gene	Primer name	Sequences	Expected product size	Source
Luciferase mRNA	*ref* forward primer(1)	5'-TACAACACCCCAACATCTTCGA-3'	67 bp	Johnson et al., 2005[1]
	ref reverse primer(2)	5'-GGAAGTTCACCGGCGTCAT-3'		
bphA	*bphA* 463f	5'-CGCGTSGMVACCTACAARG-3'	211 bp	Petrić et al., 2011[2]
	bphA 674r	5'-GGTACATGTCRCTGCAGAAYTGC-3'		

(38) Nucleotide position 1691, (2) Nucleotide position 1758

Table S3. qPCR reagent mixture composition and parameters in accordance with MIQE guidelines.

Target gene	Primer conc (μM)	Bovine serum albumin (μg)	Template	qPCR linear range, gene copies/reaction	qPCR efficiency	Y-intercept
Luciferase mRNA	0.1	0.5	2 μL cDNA	$30 – 3 \times 10^7$	102.14%	34.65
bphA	1	2	10 ng DNA or 2 μL cDNA	$30 – 3 \times 10^7$	96-100%	38.22 – 38.70

qPCR procedures. Each reaction (20 μL) contained 10 μL Power SYBR Green PCR Master Mix (Life Technologies, Warrington, UK), 0.1–1 μM primers, 0.5–2 μg bovine serum albumin (New England Biolabs, Ipswich, MA), and template DNA (Table S3). An ABI 7000 Sequence Detection System (Applied Biosystems, Grand Island, NY) was used with the following thermocycling conditions: 10 min at 95℃, 40 cycles at 95℃ (15 s) and 60℃ (1 min), and a dissociation step. A *bphA* standard curve was prepared in triplicate (Table S3); for luciferase mRNA, standard DNA templates were gBlock fragments (Integrated DNA Technologies, Coralville, IA). Melt curve analysis indicated single peaks in standards and samples for *bphA* (85°C) and luciferase (81°C).

The entire qPCR procedure was conducted in a biosafety cabinet (BSC). All supplies such as 8-channel pipettes and repeater were wiped with 70% ethanol before being brought into the BSC. All supplies were irradiated under UV light in the BSC for 15 minutes. All reagents were thawed in the ice basket, including Power SYBR green master mix, primers, DNA samples, and DNA standards, to keep the reagent activity from decreasing. Known amounts of *bphA*, cloned into the 2.1-TOPO vector as described below, were used to prepare the standard DNA. Concentrated samples were diluted to 5-10 ng/μL using qPCR-grade water prior to analysis on instrument.

Luciferase mRNA recovery efficiency was determined as the ratio of luciferase gene copies from qPCR analysis over spiked luciferase mRNA gene copies (6.08×10^9 cDNA per ng luciferase mRNA, based on dsDNA). The residual *bphA* in RNA samples after DNA digestion was measured in control qPCRs using RNA as the template. Although incomplete DNA digestion could

contribute to the low level of DNA contamination, the difference between *bphA* transcript abundance in samples and RNA controls was at least four orders of magnitude (Figure S2). Transcript levels for *bphA* in samples are shown in Figure 3.

Plasmid bphA standards transformation to 2.1-TOPO vector. Two microliters of plasmid *bphA* standards were gently mixed with a vial of Chemically Competent *E. coli* cells provided in TOPO TA Cloning kit (Life Technologies, Warrington, UK). After incubating on ice for 10 minutes, cells were provided with heat-shock at 42˚C water bath for exactly 30 seconds and then immediately transferred to ice. Afterwards, 250 µL of room temperature S.O.C. medium (provided by TOPO TA Cloning kit) was added into the cells and shaken horizontally (200rpm) at 37˚C for 1 hour. 20 µL of *E. coli* culture were spread on a prewarmed LB plate containing 50 µg/mL kanamycin and incubated overnight at 37˚C. 10 single colonies were picked from the plate and cultured in LB medium containing 50 µg/mL kanamycin overnight to grow enough *E. coli* cells for further plasmid DNA extraction which was performed based on PureLink Quick Plasmid Miniprep Kit (ThermoFisher Scientific, Waltham, MA) instructions. To verify the transformed plasmid, polymerase chain reaction (PCR) and DNA sequencing were performed to obtain the whole plasmid DNA sequences. LB medium containing 50 µg/mL kanamycin was prepared by adding 10 g tryptone, 5 g yeast extract, and 10 g NaCl in 1L DI water, and then pH was adjusted to 7. Liquid was autoclaved for 30 minutes at 15 psi, and kanamycin was added after the solution cooled to about 55˚C. For LB plate containing 50 µg/mL kanamycin, additional 15g/L agar was added into LB medium before autoclaving.

Custom probes for LB400 rRNA removal. LB400 ribosomal RNA fasta files were downloaded from the Silva database (https://www.arb-silva.de/).

RNA-seq data analysis. The first base of every read was trimmed by fastp program since the per base sequence quality was low (<20). For HISAT2, paired-end data from single interleaved dataset was selected for the library setting, and reverse (RF) was specified for strand information. For FeatureCount, GFF feature types filter was set as “gene” instead of “exon”.

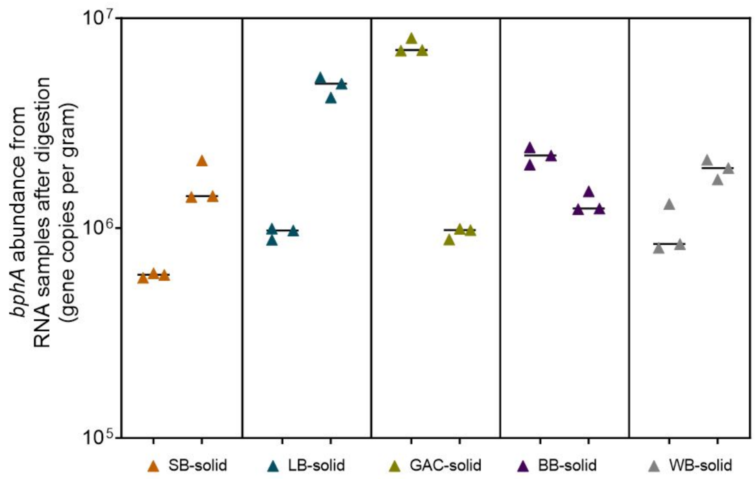

Figure S2. Residual DNA in RNA samples of attached LB400 cells in different black carbon addition systems after DNA digestion. The unit was based on wet weight of BCs. SB: small corn biochar, LB: large corn biochar, GAC: granular activated carbon, BB: bamboo biochar, and WB: wood biochar.

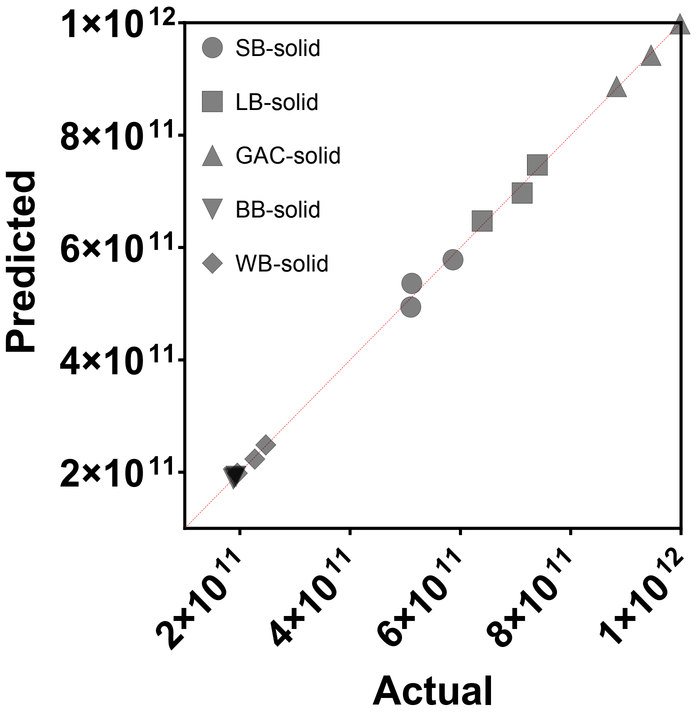

Figure S3. Shapiro-Wilk normality test of cDNA of solid samples from all five black carbon materials. SB: small corn biochar, LB: large corn biochar, GAC: granular activated carbon, BB:

bamboo biochar, and WB: wood biochar. P-values for each sample set are larger than 0.1, indicating that distribution of data was not significantly different from a normal distribution.

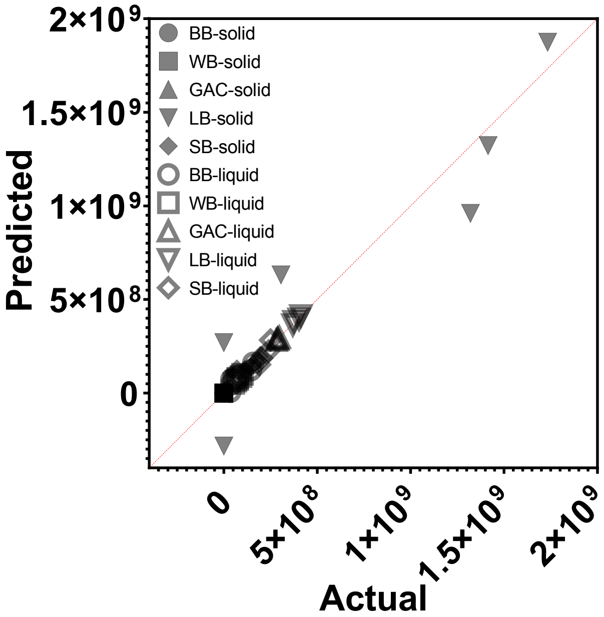

Figure S4. Shapiro-Wilk and Kolmogorov-Smirnov normality test of DNA of liquid and solid samples from all five black carbon materials. SB: small corn biochar, LB: large corn biochar, GAC: granular activated carbon, BB: bamboo biochar, and WB: wood biochar. P-values for each sample set are larger than 0.05 except GAC-solid ($p=0.0044$), indicating that distribution of data was not significantly different from a normal distribution.

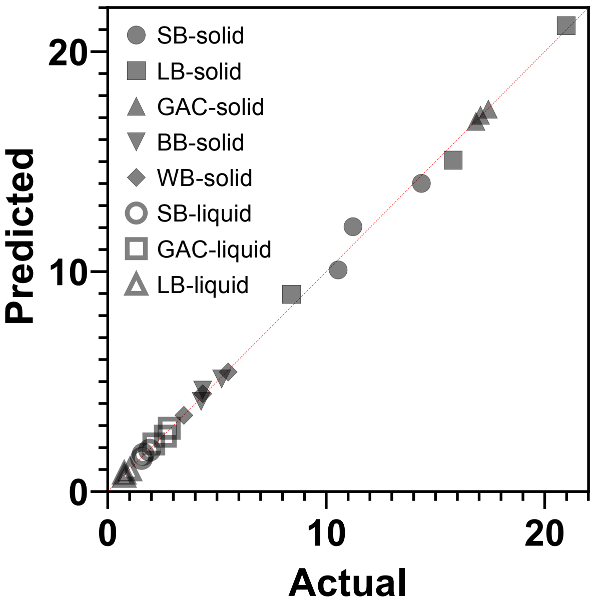

Figure S5. Shapiro-Wilk normality test of transcript per gene ratio of liquid and solid samples from different black carbon materials. SB: small corn biochar, LB: large corn biochar, GAC: granular activated carbon, BB: bamboo biochar, and WB: wood biochar. P-values for each sample set are larger than 0.05 except LB-liquid (p=0.0158).

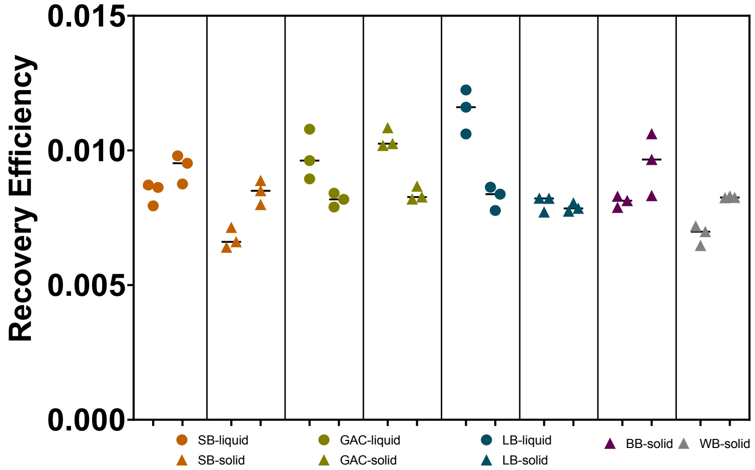

Figure S6. cDNA recovery efficiency of suspended and attached LB400 cells in different black carbon addition systems calculated based on luciferase mRNA control. SB: small corn biochar, LB: large corn biochar, GAC: granular activated carbon, BB: bamboo biochar, and WB: wood biochar.

S1.5 Biphenyl and benzoate sorption

Benzoate and biphenyl sorption by BCs and the solid-water partition coefficient (K_d). Benzoate (43 mg) or biphenyl (22.8 mg or 3 mg) was mixed in K1 media (30 mL) with or without BCs and shaken (150 rpm) for 6 days. Aqueous concentrations of biphenyl were measured over time. Aqueous biphenyl concentrations reached equilibrium after 2 days (Figure S7). Liquid samples were collected after 6 days, and analytical duplicates were quantified for each BC group. The K_d (units: L/g) was calculated as the ratio of chemical sorption to BC to chemical remaining in the aqueous phase at equilibrium. K_d = ((initial mass (mg)– mass remaining in aqueous phase after equilibrium (mg)) / mass of sorbent (g))/ aqueous chemical concentration (mg/L).

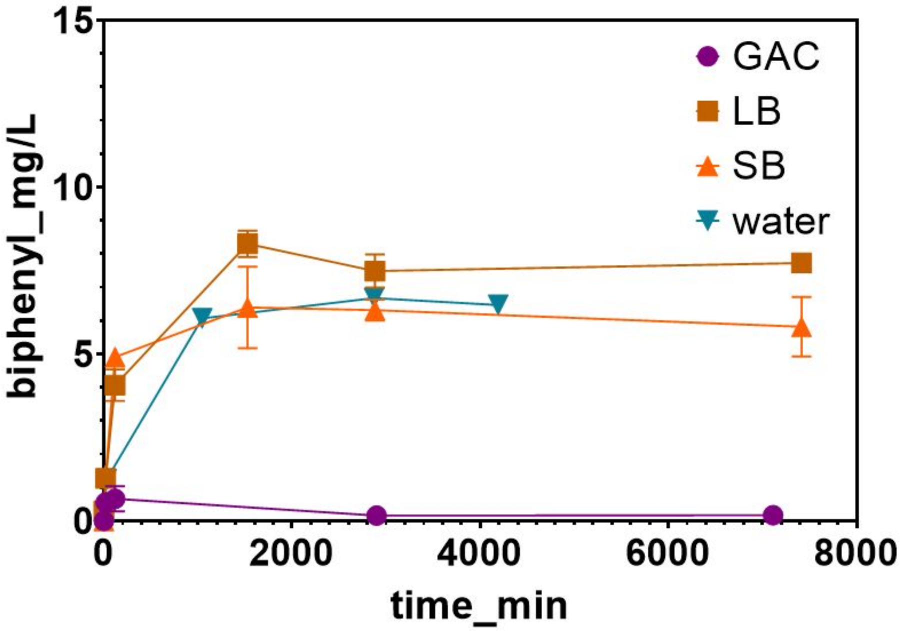

Figure S7. Preliminary results of biphenyl sorption by black carbon. Aqueous concentration of biphenyl when 3 mg biphenyl was added in liquid media or sorbed by various types of black carbons over time. LB: large corn kernel biochar, SB: small corn kernel biochar, and GAC: granular activated carbon (n=2). The error bars represent the standard deviation of treatment duplicates.

S1.6 Biphenyl, Benzoate and Acetate Analysis

Biphenyl extraction. Biphenyl was extracted from liquid samples (2 mL) via liquid-liquid extraction. Liquid samples were collected by glass analytical syringe into glass vials. Hexane was added at a 1:1 ratio (hexane:sample, v/v), followed by vortexing (10 seconds) and centrifugation (3 min at 1550×g). Supernatant was transferred to new glass vials for silica column filtration (Figure S8). Each hexane extract was filtered through a silica column and rinsed three times with hexane, with all filtrates collected in TurboVap tubes. The sample extract was concentrated to ~0.5mL using TurboVap II Concentration Workstation (Biotage, Uppsala, Sweden). The concentrated extract was transferred to crimp-top glass autosampler vials for gas chromatography.

Silica column filtration setup for biphenyl extraction. A silica column was assembled using glass wool, silica gel, and acidic silica gel, respectively, in a 23 cm disposable Pasteur pipette placed in a pipette holder. Glass wool was packed into the funnel position, where the edge and the center of cotton were sturdy and flat. Less than 1 cm silica gel was poured through the funnel, followed by acidic silica gel until there was about 2 cm away from the top of the pipette. All columns were conditioned by running through hexane for two times.

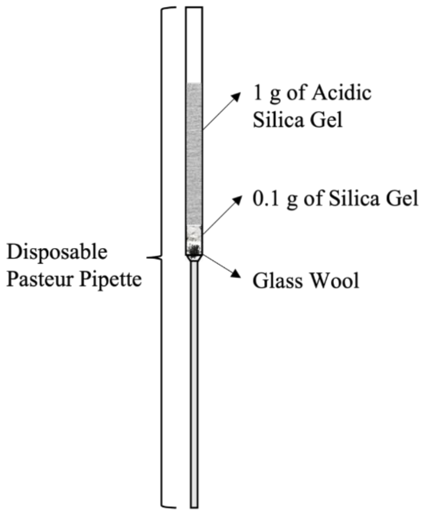

Figure S8. Display of silica column setup used to filter biphenyl extracts.

Biphenyl measurement. Extracted samples were run on a Hewlett Packard Model 6890 Gas Chromatograph equipped with 5973 Turbo Pump Mass Selective Detector (GC-MS). A Restek Rtx-VMS capillary column (30 m × 0.25 mm ID, 1.4 µm film thickness) was used with helium as the carrier gas (20 mL/min with split ratio of 10). The initial temperature GC oven temperature was 60°C (hold for 0.5 min), then ramped to 225°C (30°C/min) and hold for 2 mins. MSD electron ionization source was set to 280°C.

Benzoate measurement. Benzoate was quantified using high performance liquid chromatography (HPLC, Agilent 1100 series, Hewlett Packard, Palo Alto, CA) with Diode-Array Detection (DAD) and separated on a Sprite Targa reverse-phase C18 column (40 mm × 2.1 mm ×5 µm) with a PEEK

guard column.[3] The HPLC operating mode was isocratic and an injection volume of 10 μL was loaded onto the column at ambient temperature. The mobile phase contained ammonium acetate buffer (90%) and acetonitrile (10%) at a flow rate of 0.8 mL/min. Sample data collection time was 8 mins per sample with UV detection at 225 nm. Ammonium acetate buffer was prepared with 0.3 g ammonium acetate adding in 900 mL DI water, followed by the addition of glacial acetic acid adjusting pH to 3.9. The buffer solution was then transferred to 1 L volumetric flask and filtered through 0.22 μm nylon filter. Method detection level (MDL) was 0.48 mM. Desired MDL was 3.14 times standard deviation of seven replicates of benzoate solution. The recovery efficiency was within the range of 50-150%, and replicate measurements were in the range of one to five times the MDL. MDL determination followed the instructions of Standard Methods for the Examination of Water and Wastewater.[4]

Acetate measurement. Acetate concentrations in liquid samples were measured by Ion Chromatography (Dionex ICS-2100) equipped with AS11-HC (250×4 mm) anion exchange column and an AG11 guard column. The column temperature was 30℃, the cell temperature was 35℃, and the suppressor was set at 41 mA. The effluent flow rate was 1.1 mL/min. The eluent gradient was KOH ramp from 1 to 60 mM with a total run time of 20 mins. Method detection level was 0.017 mM. Desired MDL was 3.14 times standard deviation of seven replicates of acetate solution. The recovery efficiency was within the range of 50-150%, and replicate measurements were in the range of one to eight times the MDL. Determination also followed the instructions of Standard Methods for the Examination of Water and Wastewater.[4]

S2. Supplemental Results

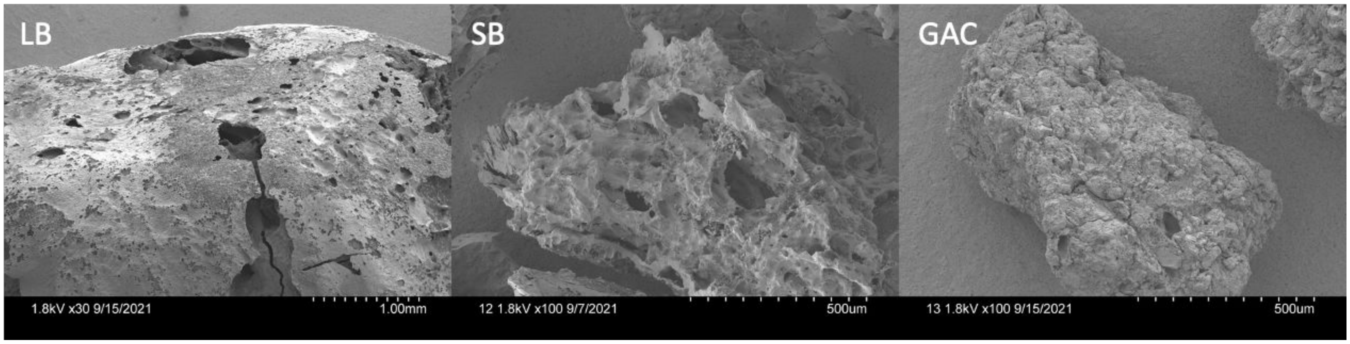

S2.1 Black Carbon Characterization

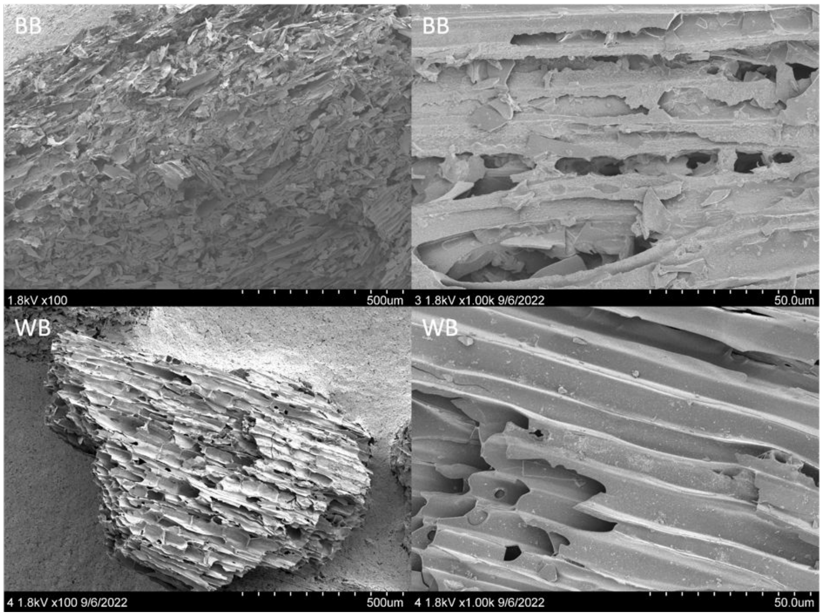

Figure S9. SEM imaging of black carbon surfaces: BB represents bamboo biochar, WB represents wood biochar, LB represents large corn biochar, SB represents small corn biochar, and GAC represents granular activated carbon.

Table S4. Spearman correlation between BC properties and *bphA* abundance in attached cells.

	bphA per gram vs. K_d	*bphA* per gram vs. Mesoporous volume	*bphA* per gram vs. Mesopore size	*bphA* per gram vs. BET surface area	*bphA* per gram vs. Total porous volume	*bphA* per gram vs. BC pH
Spearman r	-0.6	-0.6	-0.9	-0.6	-0.5	-0.6669
P value (two-tailed)	0.35	0.35	0.0833	0.35	0.45	0.2667

Significant? (alpha = 0.1)	No	No	Yes	No	No	No

S2.2 Viability and Activity of LB400 Attached Cells

Table S5. Summary of live to dead cell ratios on corn kernel biochar and GAC surfaces growing under 5 mM biphenyl crystal for 10 days.

	Cell Number	Live to Dead Ratio	Average Distance to Surface (μm)
GAC 1_green	4746		1.98
GAC 1_red	3319	1.43	1.36
GAC 2_green	1249		2.51
GAC 2_red	1022	1.22	0.60
GAC 3_green	1152		-0.11
GAC 3_red	1242	0.93	0.12
SB 1_green	1152		1.69
SB 1_red	269	4.28	1.67
SB 2_green	860		6.58
SB 2_red	101	8.51	2.83
SB 3_green	500		5.94
SB 3_red	110	4.55	0.39

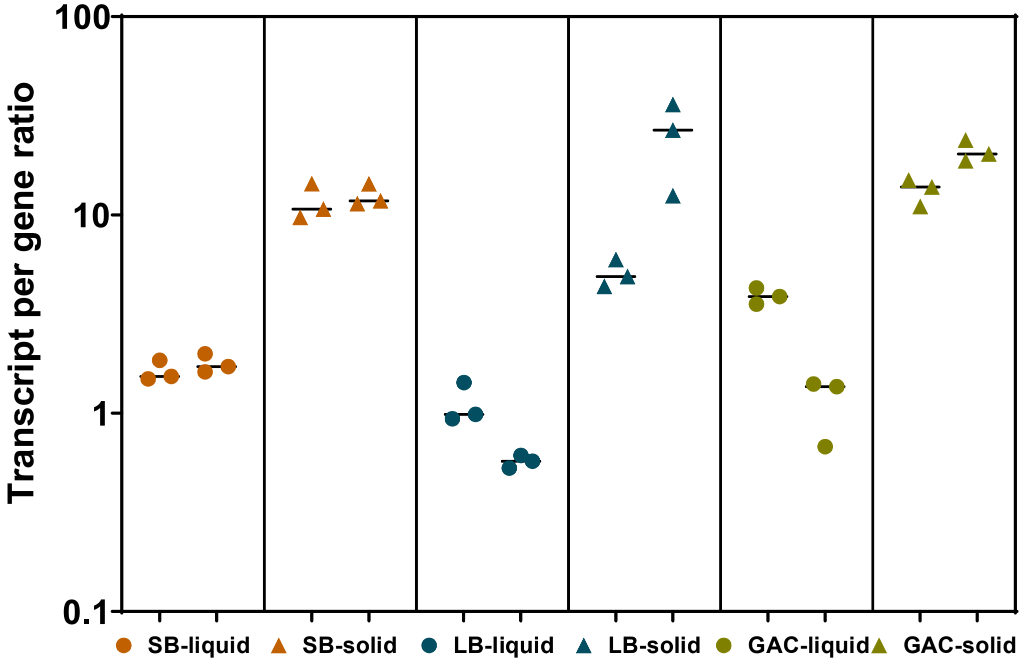

Figure S10. Transcript per gene ratio of suspended and attached LB400 cells in different black carbon addition systems. SB: small corn biochar, LB: large corn biochar, and GAC: granular activated carbon. Liquid phase represents suspended cells, and solid phase represents attached cells.

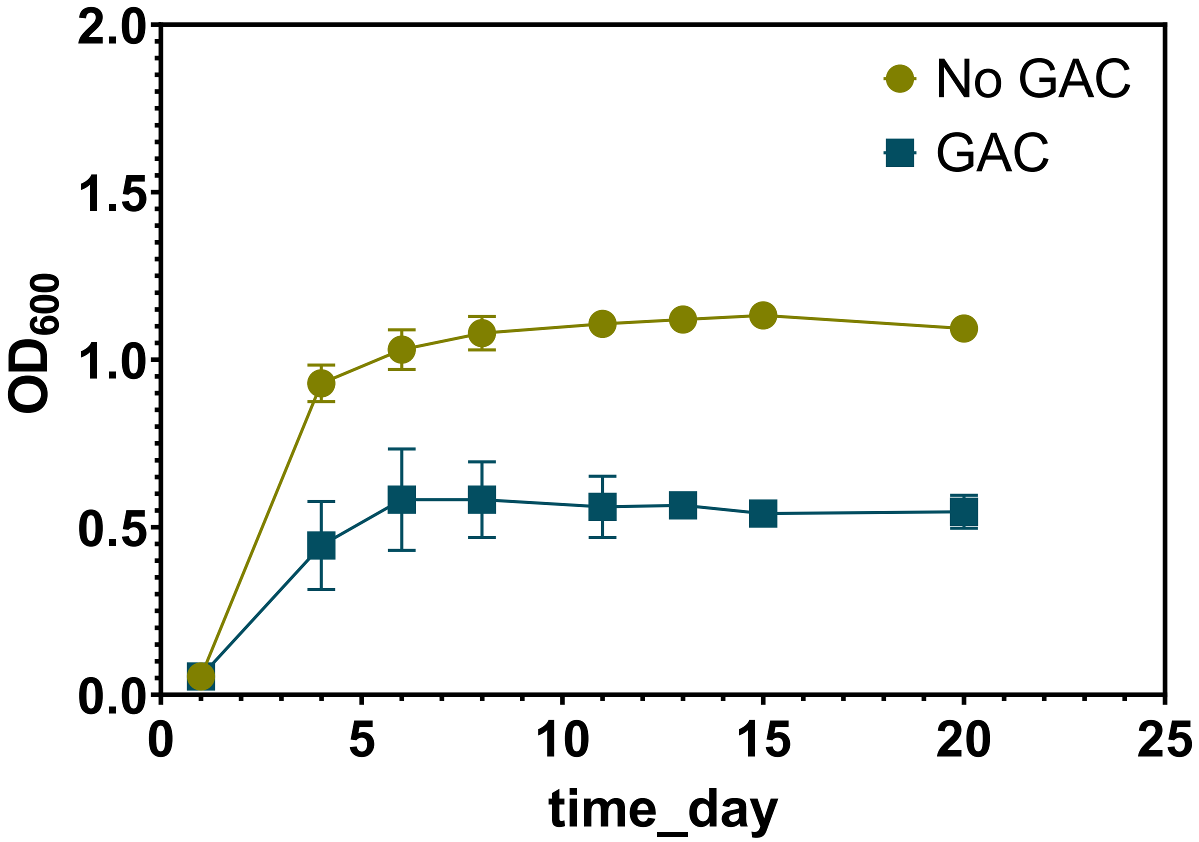

Figure S11. Growth curve of LB400 with or without GAC using 5 mM biphenyl as sole carbon source. Error bars represent the standard deviation of biological duplicates.

S2.3 RNA-seq of attached and suspended LB400 cells

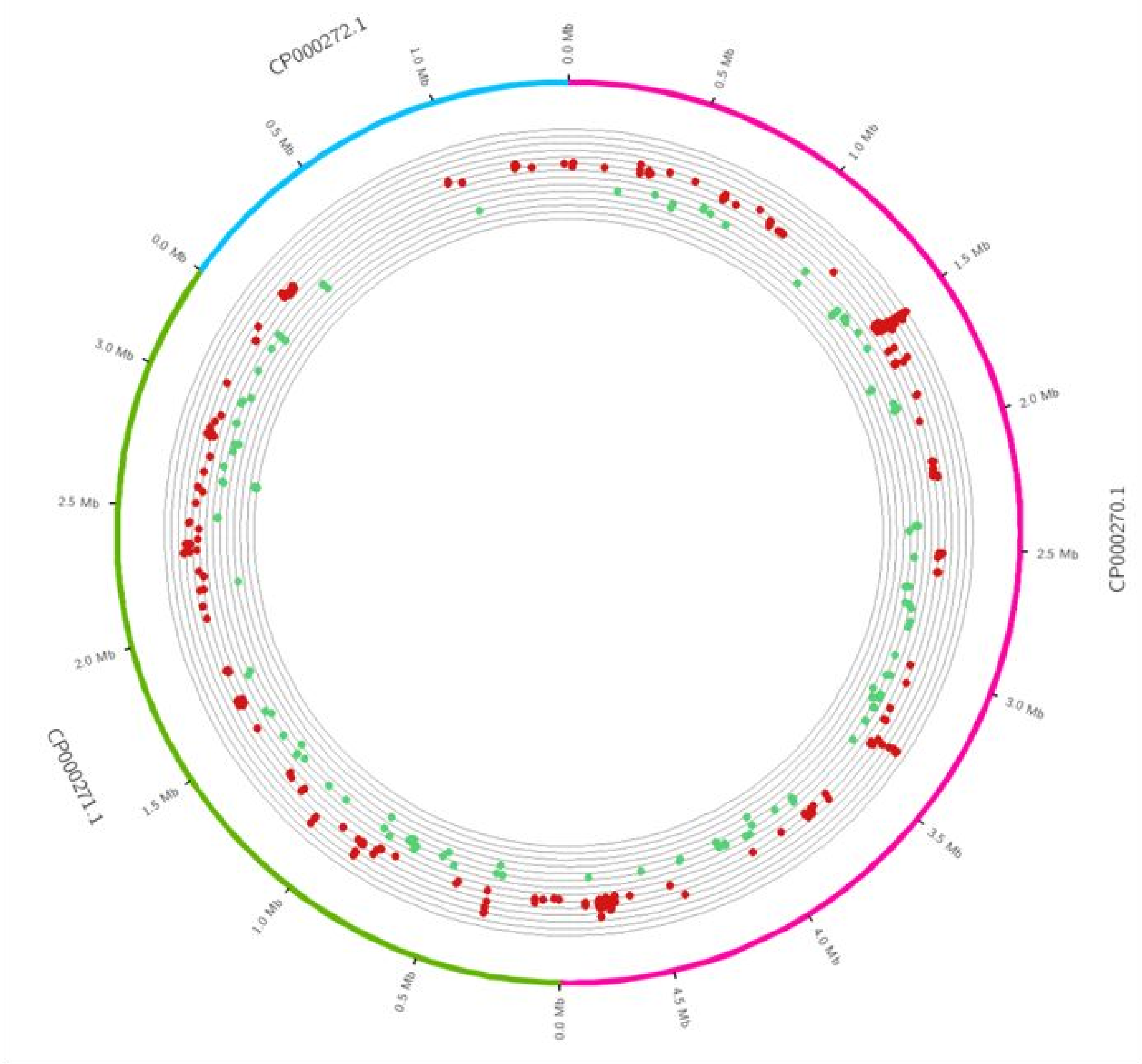

Figure S12. Differential gene expression between attached and suspended LB400 cells growing in the presence of BC feedstocks (LB, SB, and GAC). The genome region in pink (Genbank Accession No. CP000270.1) is chromosome 1, the region in green (Genbank Accession No. CP000271.1) is chromosome 2, and the region in blue (Genbank Accession No. CP000272.1) is the megaplasmid. Upregulated genes are shown in red, while downregulated genes are shown in green. Selected differentially expressed genes pass the threshold of log2(FC)>|±1|, p-adj<0.05. Each grey ring represents one log2(FC), where the innermost ring is log2(FC)=-7 and the outermost ring is log2(FC)=7.

Table S6. *Bph* pathway related genes of differential gene expression comparison between attached and suspended LB400 cells.

Gene ID	log2(FC)	P-adj	Gene and gene product
Bxe_C1186	1.36	1.09×10^{-8}	*bphD*
Bxe_C1187	1.53	9.60×10^{-9}	*bphI*
Bxe_C1188	1.55	6.94×10^{-08}	*bphJ*
Bxe_C1189	1.69	4.30×10^{-7}	*bphH*
Bxe_C1190	1.87	5.64×10^{-10}	*BphK*
Bxe_C1191	1.67	6.94×10^{-08}	*bphC*
Bxe_C1192	1.66	2.02×10^{-06}	*bphB*
Bxe_C1193	1.62	0.000149	*bphA4*
Bxe_C1194	1.82	4.47×10^{-5}	*bphA3*
Bxe_C1195	1.57	8.51×10^{-5}	*bphX* family protein
Bxe_C1196	1.68	3.17×10^{-7}	*bphA2*
Bxe_C1197	1.54	2.03×10^{-6}	*bphA1*

Table S7. *Box* pathway related genes of differential gene expression comparison between attached and suspended LB400 cells.

Gene ID	log2(FC)	P-adj	Gene and gene product
Bxe_A1419	5.03	6.65×10^{-36}	benzoate-CoA ligase family protein, *BCL*
Bxe_A4554	5.11	8.49×10^{-40}	Unknown protein (box pathway), potential *ADH*
Bxe_A1422	5.00	1.17×10^{-29}	*boxC*
Bxe_A1423	5.18	8.18×10^{-41}	*boxB*
Bxe_A1424	4.47	1.42×10^{-42}	*boxA*

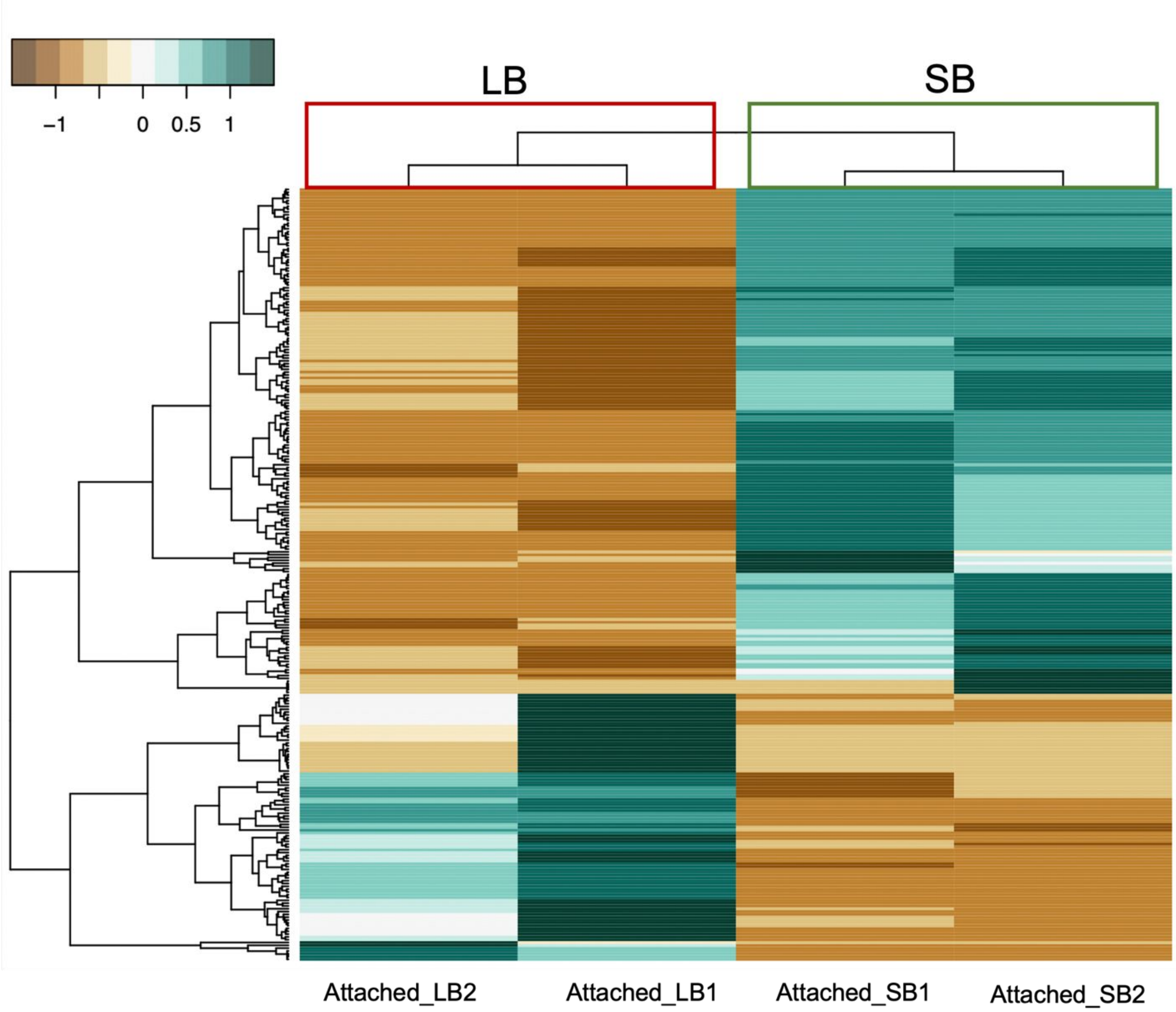

Figure S13. Differential gene expression heatmap of LB400 cells attached to either SB or LB. Green bars represent upregulated genes while yellow bars represent downregulated genes. Both heatmaps were plotted by using Z-scores, standardized based on row, and describe the significant gene expression level of selected genes passing the threshold (Log2 (FC)> |±1|, adjusted $p<0.05$). The Z-score is the number of standard-deviations that a value was away from the mean of all the values in the same group. Hierarchical clustering was based on the Euclidean distance of each row and demonstrated the similarity of different genes. SB: small corn biochar and LB: large corn biochar. Branches on the left depicted the cluster of selected genes and branches on the top demonstrated the cluster of samples.

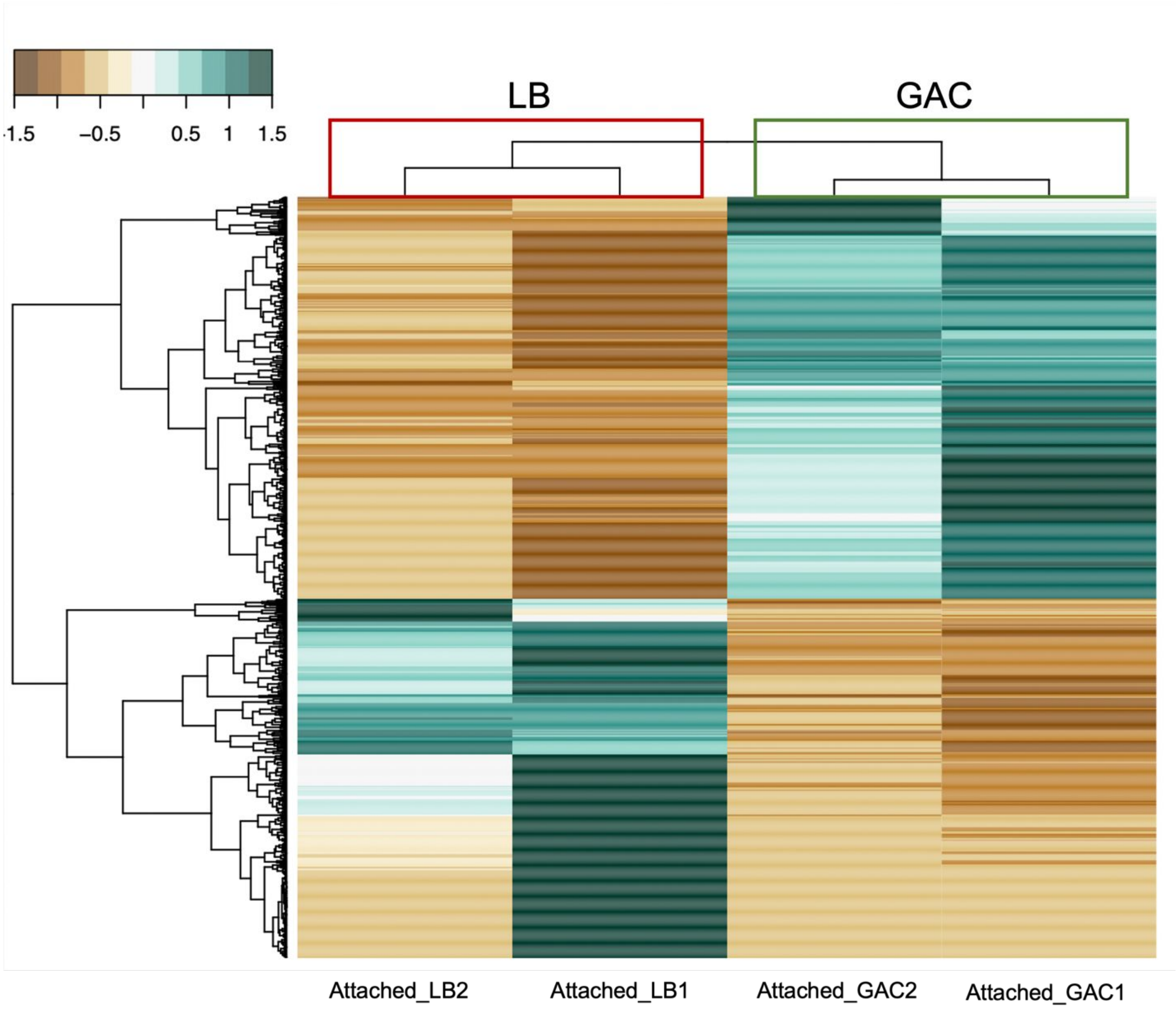

Figure S14. Differential gene expression heatmap of LB400 cells attached to either LB or GAC. Green bars represent upregulated genes while yellow bars represent downregulated genes. Both heatmaps were plotted by using Z-scores, standardized based on row, and describe the significant gene expression level of selected genes passing the threshold (Log2 (FC)> |±1|, adjusted $p<0.05$). The Z-score is the number of standard-deviations that a value was away from the mean of all the values in the same group. Hierarchical clustering was based on the Euclidean distance of each row and demonstrated the similarity of different genes. LB: large corn biochar and GAC: granular activated carbon. Branches on the left depicted the cluster of selected genes and branches on the top demonstrated the cluster of samples.

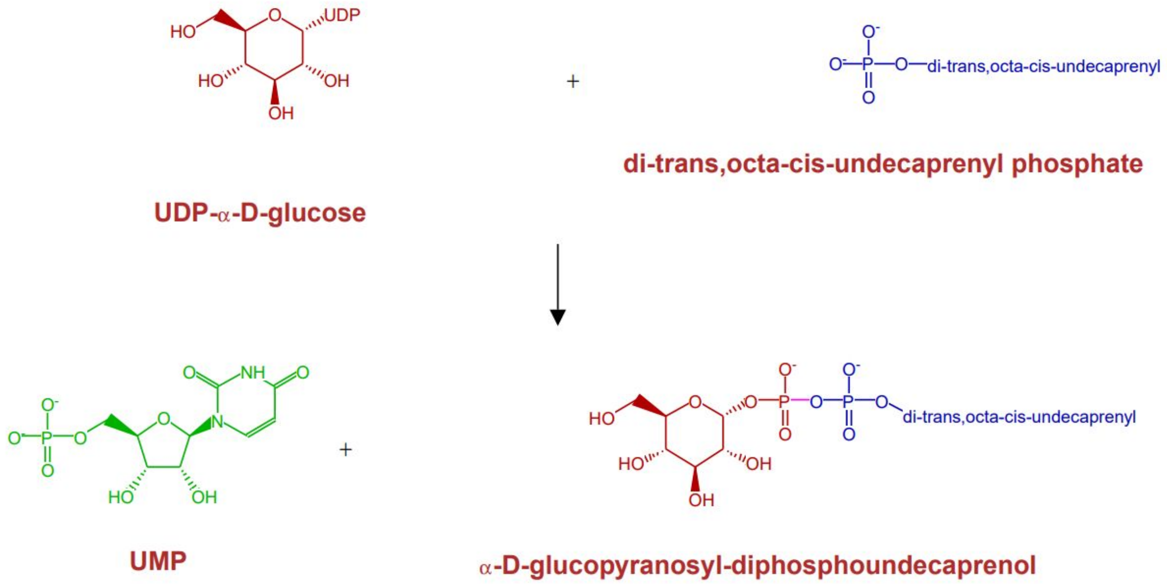

Figure S15. Extracellular polymeric substances (EPS) related pathway (EPS pathway I).[5-8]

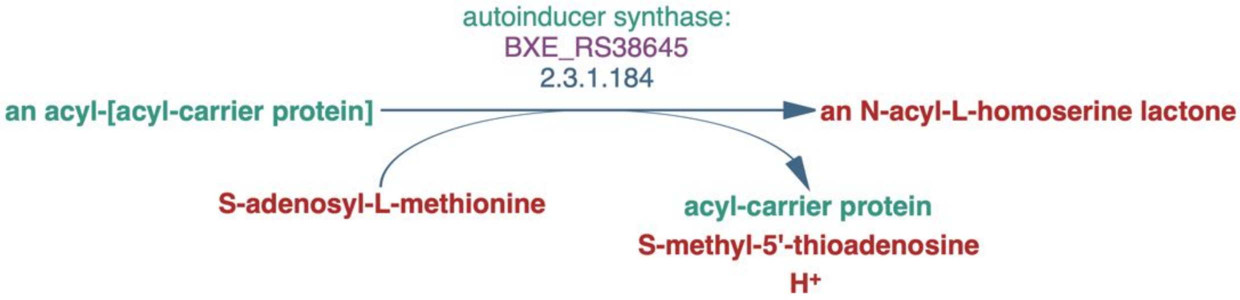

Figure S16. N-acylhomoserine lactone (AHL) production pathway.[5-7, 9]

Table S8. Genes related to EPS pathways (EPS pathway I) of differential gene expression comparison between LB and GAC surfaces.

GeneID	log2(FC)	p-adj	Gene product	Note
Bxe_A2241	4.33	0.0085	Putative glycosyl transferase, family 2, involved in cell wall biogenesis	UDP-glucose, EPS precursor, is involved in glycosyltransferase reactions. Bacterial capsule biosynthesis
Bxe_A2243	5.67	0.036	polysaccharide (EPS I) exporter, outer membrane auxiliary (OMA) protein family	EPS biosynthesis and/or export
Bxe_A2245	6.01	0.042	UDP-glucose 6-dehydrogenase	Biosynthesis of UDP-glucuronic acid, nucleotide donors.
Bxe_A2246	7.49	0.019	Undecaprenyl- phosphate glucose phosphotransferase	glucose 1-phosphate

Table S9. Genes related to EPS production pathway (EPS pathway II) of differential gene expression comparison between LB and GAC surfaces and SB and GAC surfaces.

	LB vs GAC		SB vs GAC			
GeneID	log2(FC)	p-adj	log2(FC)	p-adj	Gene product	Note
Bxe_B1722	2.80	0.017	1.87	7.10×10^{-5}	EPS biosynthesis protein	EPS biosynthesis
Bxe_B1725	2.58	0.038	1.46	0.00094	glycosyltransferase family 2 protein	bacterial capsule biosynthesis
Bxe_B1726	2.51	0.026	1.00	0.0067	polysaccharide biosynthesis tyrosine autokinase	synthesis and assembly of capsular and EPS
Bxe_B1727	2.94	0.026	-	-	low molecular weight phosphotyrosine protein phosphatase	protein tyrosine synthesis
Bxe_B1728	2.71	0.021	1.08	0.0063	sugar ABC transporter substrate-binding protein	transport sugar into cytosol

Table S10. AHL production pathway related genes of differential gene expression comparison between SB and GAC surfaces.

GeneID	log2(FC)	p-adj	Gene and gene product	Note
Bxe_C0415	2.80	0.0016	*xenR2*, acyl-homoserine lactone synthase	autoinducer synthase
Bxe_C0416	-1.08	0.00286	*xenI2*, luxR transcriptional regulator	AHL receptor in QS system
Bxe_C0417	2.49	1.78×10^{-13}	3-oxoacyl-ACP synthase	form acyl-ACP
Bxe_C0418	2.43	1.12×10^{-13}	3-oxoacyl-ACP synthase	form acyl-ACP
Bxe_C0423	2.78	0.020	3-oxoacyl-ACP synthase	form acyl-ACP
Bxe_C0424	3.09	0.0010	3-oxoacyl-ACP synthase	form acyl-ACP
Bxe_C0425	3.34	1.70×10^{-20}	acyl carrier protein	ACP
Bxe_C0419	3.00	5.54×10^{-18}	GNAT family N-acetyltransferase	chemical modification of the amine group (e.g., SAM) with an acetyl group
Bxe_C0421	3.05	0.00086	pyridoxal-dependent decarboxylase, exosortase A system-associated	Potential SAM to S-methyl-5'-thioadenosine (MTA)
Bxe_C0422	2.88	0.00048	acyl-CoA ligase	long chain fatty acid to fatty acyl CoA
Bxe_C0426	3.08	0.00072	acyl-CoA dehydrogenase	

Bxe_C0420	3.18	7.79×10^{-22}	acyl-CoA/acyl-ACP dehydrogenase	oxidate acyl-ACP

S2.4 Biodegradation of Chemicals by Black Carbon Attached LB400

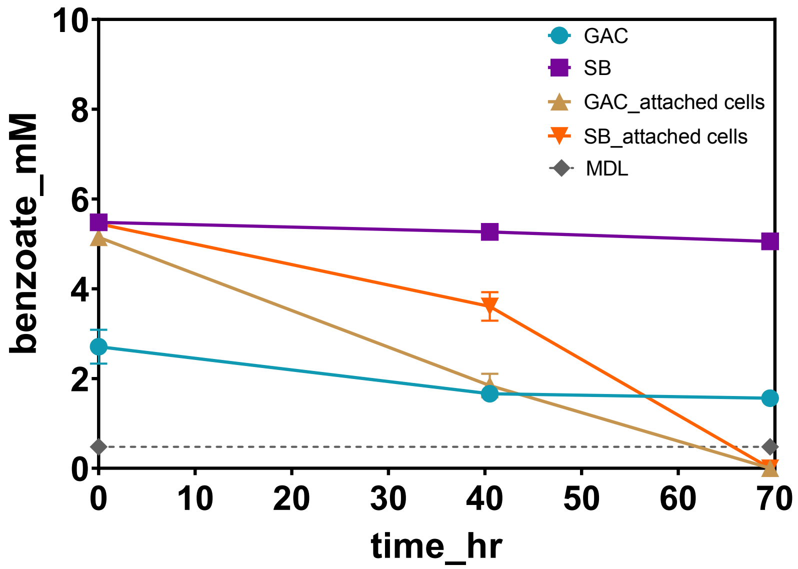

Figure S17. Benzoate degradation in the liquid phase for both cells attached on SB and GAC and benzoate sorption by plain SB and GAC (n=2). Attached cells were grown under 5 mM biphenyl for 10 days before testing benzoate degradation. The initial sampling time was immediately after BCs (with and without attached cells) and benzoate were added and mixed. The initial benzoate concentration for GAC (without attached cells) was lower than other groups because benzoate was rapidly sorbed by GAC. Attached cells may have affected benzoate sorption efficiency in the GAC_attached cells treatment. SB: small corn biochar and GAC: granular activated carbon. Error bars represent the standard deviation of biological duplicates. MDL represents method detection limit of benzoate.

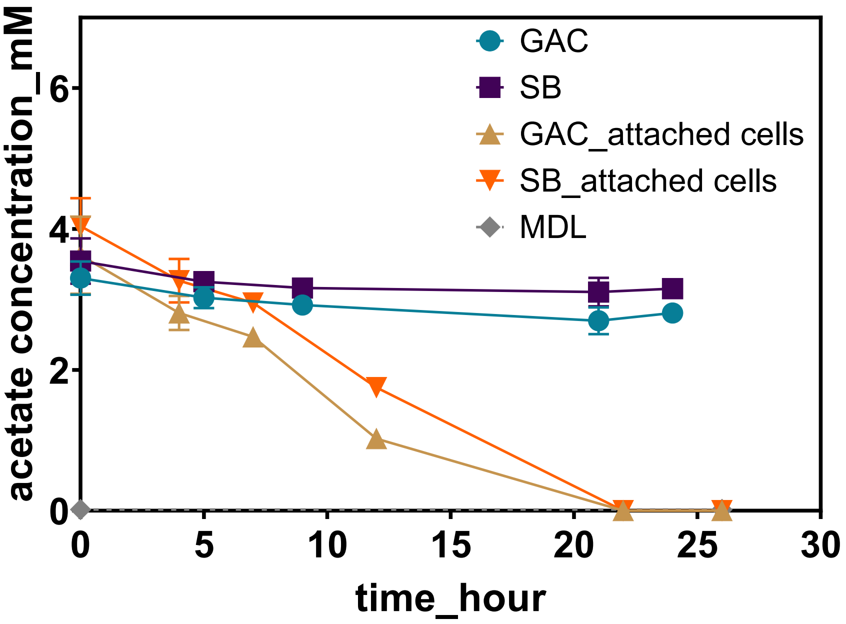

Figure S18. Acetate degradation in the liquid phase for both cells attached on SB and GAC and acetate sorption by plain SB and GAC (n=4). Attached cells were grown under 5 mM biphenyl for 10 days before testing acetate degradation. SB: small corn biochar and GAC: granular activated carbon. Error bars represent the standard deviation of biological duplicates. MDL represents method detection limit of acetate.

S2.5 <u>Soaking Black Carbon with Biphenyl or Benzoate</u>

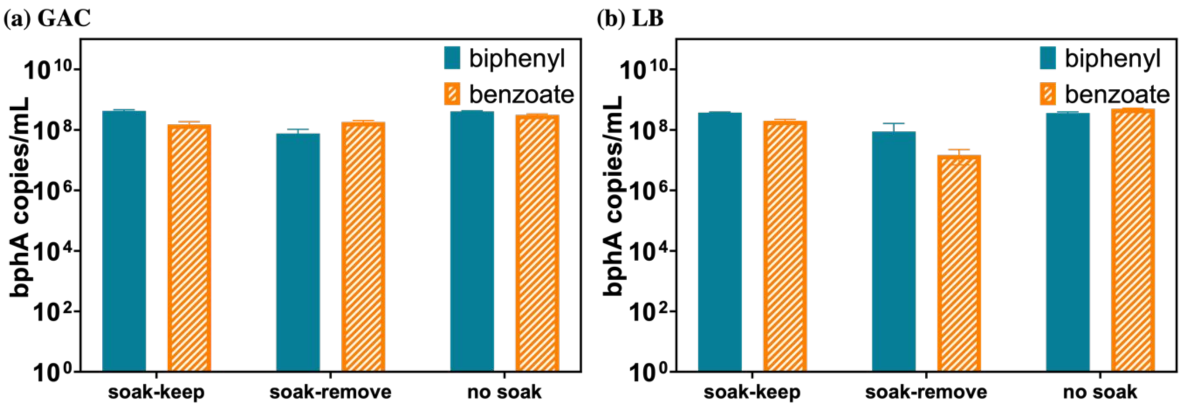

Figure S19: *BphA* gene abundance in liquid medium with the addition of GAC or LB with either 43 mg benzoate or 150 mg biphenyl at 10-day. "Soak-keep" and "soak-remove" represent carbon source was washed away from the liquid media or not after soaking. "No soak" represents control groups. LB: large corn biochar and GAC: granular activated carbon. All controls and treatments were done by duplicates. Error bars represent the standard deviation of biological duplicates.

References

1. Johnson, D. R.; Lee, P. K.; Holmes, V. F.; Alvarez-Cohen, L., An internal reference technique for accurately quantifying specific mRNAs by real-time PCR with application to the tceA reductive dehalogenase gene. *Applied and environmental microbiology* **2005,** *71*, (7), 3866-3871.
2. Petrić, I.; Hršak, D.; Fingler, S.; Udiković-Kolić, N.; Bru, D.; Martin-Laurent, F., Insight in the PCB-degrading functional community in long-term contaminated soil under bioremediation. *J Soils Sediments* **2011,** *11*, 290-300.
3. Pylypiw Jr, H. M.; Grether, M. T., Rapid high-performance liquid chromatography method for the analysis of sodium benzoate and potassium sorbate in foods. *J Chromatogr A* **2000,** *883*, (1-2), 299-304.
4. American Public Health Association; American Water Works Association, W. E. F., *Standard Methods for the Examination of Water and Wastewater*. 24th ed.; American Public Health Association: Washington, DC, 2022.
5. Caspi, R.; Billington, R.; Keseler, I. M.; Kothari, A.; Krummenacker, M.; Midford, P. E.; Ong, W. K.; Paley, S.; Subhraveti, P.; Karp, P. D., The MetaCyc database of metabolic pathways and enzymes - a 2019 update. *Nucleic Acids Res* **2019,** *48*, (D1), D445-D453.
6. Karp, P. D.; Latendresse, M.; Caspi, R., The pathway tools pathway prediction algorithm. *Stand Genomic Sci* **2011,** *5*, (3), 424-9.
7. Karp, P. D.; Latendresse, M.; Paley, S. M.; Krummenacker, M.; Ong, Q. D.; Billington, R.; Kothari, A.; Weaver, D.; Lee, T.; Subhraveti, P.; Spaulding, A.; Fulcher, C.; Keseler, I. M.; Caspi, R., Pathway Tools version 19.0 update: software for pathway/genome informatics and systems biology. *Brief Bioinform* **2015,** *17*, (5), 877-890.
8. BioCyc *Paraburkholderia xenovorans* LB400 Reaction: 2.7.8.31. https://biocyc.org/GCF_000013645/NEW-IMAGE?type=REACTION&object=RXN-11791 (Date Last Accessed: Oct 31, 2023),
9. BioCyc *Paraburkholderia xenovorans* LB400 Reaction: 2.3.1.184. https://biocyc.org/GCF_000013645/NEW-IMAGE?type=REACTION&object=2.3.1.184-RXN (Date Last Accessed: Oct 31, 2023),
